# Cost-effectiveness of respiratory syncytial virus vaccines in post-marketing settings: a systematic review and meta-analysis

**DOI:** 10.1186/s40249-026-01520-y

**Published:** 2026-11-14

**Authors:** Mengyao Mu, Yang Deng, Zhanwei Du, Yi Zhao

**Affiliations:** 1 School of Science, Harbin Institute of Technology, Shenzhen, 518055 China; 2 School of Mathematics, Harbin Institute of Technology, Harbin, 150001 China; 3 Shenzhen Center for Disease Control and Prevention, Shenzhen, 518055 China; 4 School of Public Health and Emergency Management, Southern University of Science and Technology, Shenzhen, 518055 China

**Keywords:** Cost-effectiveness analysis, Incremental net benefit, Meta-analysis, Respiratory syncytial virus vaccine, Systematic review

## Abstract

**Background:**

Respiratory syncytial virus (RSV) is a leading cause of acute lower respiratory infections, hospitalizations, and deaths, particularly among infants and older adults worldwide. This study aims to summarize cost-effectiveness evidence by pooling incremental net benefit (INB) estimates of marketed RSV vaccines.

**Methods:**

In this review, PubMed, Embase, Web of Science, and the Cochrane Library were searched through November 20, 2025. Grey literature and reference lists were also searched. Random-effects meta-analyses were conducted separately for older adults and infants. Potential sources of heterogeneity were explored using population-specific subgroup analyses and univariable meta-regressions.

**Results:**

Of the 17 studies included in the systematic review, 11 contributed to the primary meta-analysis and one additional study to sensitivity analysis only. Among older adults, the pooled INB was USD 105.05 [95% confidence interval (*CI*): USD 41.89–168.21; *I*^2^ = 93.2%]. For infants, the pooled INB was USD 5.81 (95% *CI:* USD − 35.44–47.07; *I*^2^ = 89.4%). In the European subgroup, the pooled INB among older adults was USD 123.63 (95% *CI:* USD 41.23–206.02; *I*^2^ = 89.4%), while that for infants was USD 15.00 (95% *CI:* USD − 4.33–34.34; *I*^2^ = 43.4%). Leave-one-out sensitivity analyses showed robust pooled INBs among older adults, whereas infant estimates were sensitive to study exclusion.

**Conclusions:**

At the thresholds used in the included studies, RSV vaccination might be cost-effective for older adults in post-marketing settings, while the evidence for maternal vaccination remained inconclusive. Descriptive findings suggested that age- and risk-based targeting in older adults and seasonal maternal vaccination may yield higher INB estimates. Substantial between-study heterogeneity warrants cautious interpretation of the pooled estimates and highlights the need to consider local epidemiology and implementation conditions when developing RSV vaccination policies.

**Graphical Abstract:**

**Figure:**
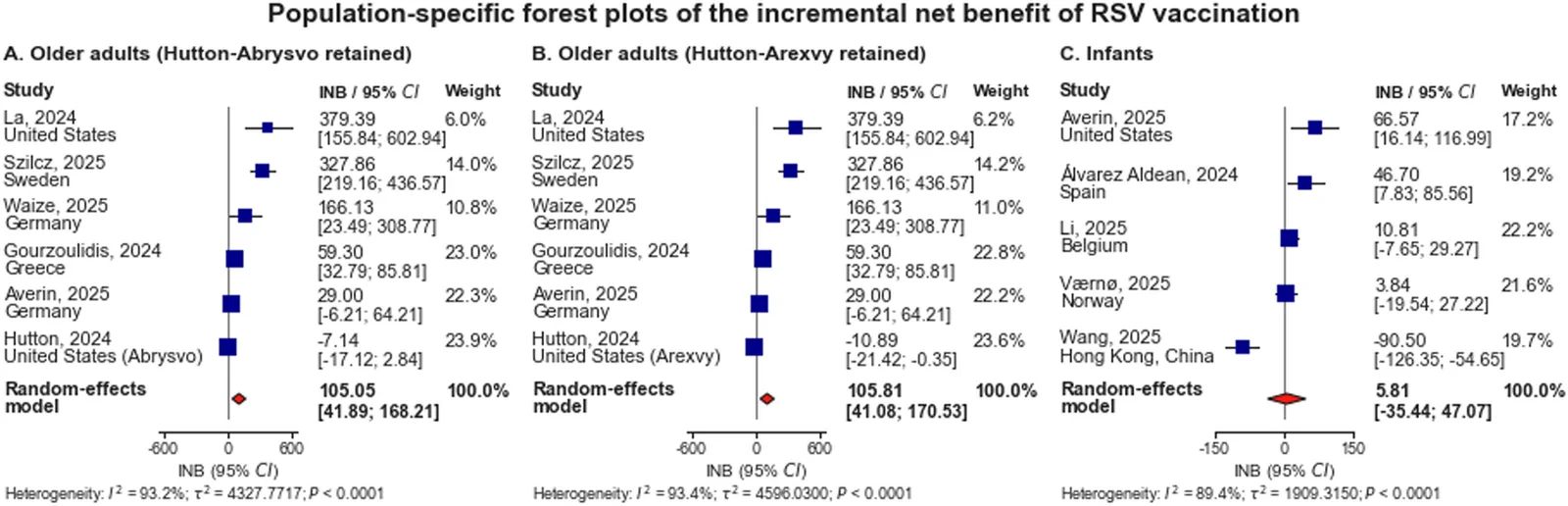

**Supplementary Information:**

The online version contains supplementary material available at https://doi.org/10.1186/s40249-026-01520-y.

## Background

Respiratory syncytial virus (RSV) is a common cause of respiratory illness among infants and older adults worldwide [1–3]. It causes approximately 33.0 million RSV-associated acute lower respiratory infection episodes, 3.6 million hospital admissions, and 101,400 RSV-attributable deaths annually among children younger than 5 years worldwide [4]. Among adults aged 60 years and older in high-income countries, RSV accounts for approximately 5.2 million cases, 470,000 hospitalizations, and 33,000 in-hospital deaths each year [5].

To mitigate the global health burden of RSV infection, the World Health Organization (WHO) has designated RSV vaccine development as a public health priority [6]. By the search cutoff, three RSV vaccines for active immunization had received regulatory approval: Arexvy [RSV prefusion F protein vaccine for older adults (RSVPreF3 OA), GlaxoSmithKline (GSK)] for older adults, Abrysvo [bivalent RSV prefusion F vaccine (RSVPreF), Pfizer] for older adults and for maternal immunization to protect infants, and mRESVIA (Moderna) for older adults [7]. Long-acting monoclonal antibodies, such as nirsevimab, are also available for infant RSV prevention but are not classified as vaccines [8, 9] and are outside the scope of this review. With the recent development and licensure of RSV vaccines, evaluating their cost-effectiveness has become increasingly important for informing immunization policy and resource allocation.

Previous systematic reviews have summarized the economic evidence on RSV vaccination across different populations and settings [10–12], but have not quantitatively synthesized findings from individual economic evaluations. In 2014, Crespo et al. proposed a framework for meta-analysis of cost-effectiveness studies using incremental net benefit (INB) as a common outcome measure [13]. This framework has been applied in several subsequent meta-analyses since 2019 [14–20]. However, to our knowledge, this framework has not yet been systematically applied to synthesize the economic evidence on RSV vaccination. The WHO Immunization and Vaccine-Related Implementation Research Advisory Committee noted in 2021 that meta-analyses of economic evaluations could support decision-making in countries with limited capacity to conduct their own cost-effectiveness studies [21]. This study aimed to assess the cost-effectiveness of marketed RSV vaccines compared with no vaccination by pooling INB estimates from economic evaluations, with separate analyses for older adults and infants.

## Methods

This review was not prospectively registered. A protocol was established prior to study selection and was provided in Supplementary material 1, together with any protocol deviations. The review was reported according to the Preferred Reporting Items for Systematic Reviews and Meta-Analyses (PRISMA) 2020, and the completed checklist was provided in Supplementary material 2. The methodological design and reporting of this review were informed by the International Society for Pharmacoeconomics and Outcomes Research (ISPOR) Good Practices Task Force Report for systematic reviews with cost and cost-effectiveness outcomes [22].

### Search strategy

We searched PubMed, Embase, Web of Science, and the Cochrane Library for studies published up to November 20, 2025, using database-specific controlled vocabulary and free-text terms related to RSV, vaccination, and economic evaluation. Search terms included “RSV”, “respiratory syncytial virus”, “vaccin*”, “immuni*”, “cost-effectiveness”, “cost-utility”, “economic evaluation”, “pharmacoeconomic*”, and “ICER” (incremental cost-effectiveness ratio). We searched grey literature through the International Network of Agencies for Health Technology Assessment (INAHTA) International Health Technology Assessment (HTA) Database, Google Scholar, Canada’s Drug Agency (CDA-AMC) reports, the WHO Institutional Repository for Information Sharing (IRIS), and Centers for Disease Control and Prevention (CDC) Advisory Committee on Immunization Practices (ACIP) meeting slides. We also screened the reference lists of eligible articles and relevant reviews for studies missed by the database searches. Detailed database and grey literature search strategies were provided in Supplementary material 3.

### Inclusion and exclusion criteria and literature screening process

Predefined inclusion and exclusion criteria were applied to identify eligible studies. For this review, a post-marketing setting was defined as a setting in which the RSV vaccine had been marketed at the time of evaluation and locally established vaccine prices were available from official or recognized pricing sources. Studies were eligible if they met the following criteria: (1) evaluated the cost-effectiveness or cost-utility of RSV vaccines in post-marketing settings, as defined above; (2) compared vaccination with no vaccination; and (3) were published in English. Studies were excluded if they met any of the following conditions: (1) focused solely on the cost of illness; (2) evaluated hypothetical vaccine prices or maximum cost-effective price-per-dose estimates; (3) lacked a standalone vaccine-only arm; (4) provided insufficient information to derive an INB; or (5) were conference abstracts or review articles. Title and abstract screening, followed by full-text assessment, was conducted independently by two reviewers (M.M. and Y.D.). Disagreements were resolved through discussion with a third reviewer (Z.D.).

### Data extraction and quality assessment

Data were extracted on study and intervention characteristics, including vaccine name, vaccine price, coverage rate, modeling type, time horizon, study perspective, outcome measures, model population size, discount rate, country income level, target population, and funding source. For each study, ICERs, incremental costs (ΔC), incremental effects (ΔE), and willingness-to-pay (WTP) thresholds were also extracted. Probabilistic sensitivity analysis (PSA) scatter plots of ΔC and ΔE were digitized using WebPlotDigitizer version 4.4 [23].

Risk of bias was assessed using the modified Economic Evaluation Bias (ECOBIAS) checklist, which contains 22 items [24]. Each item was rated as yes, partly, unclear, no, or not applicable. Three key items—limited sensitivity analysis bias, wrong model bias, and bias related to treatment effects—were used for overall risk-of-bias classification. Studies rated “yes” on all three items were classified as having a low risk of bias; those with one or more “partly/unclear” ratings were classified as moderate risk; and those with at least one “no” rating were classified as high risk. Two authors (M.M. and Y.D.) independently extracted data and assessed risk of bias. Disagreements were resolved through discussion with a third author (Z.D.).

### Outcome of interest

The primary outcome was the INB of RSV vaccination compared with no vaccination. Depending on the available data, INB was calculated using Eq. (1) or (2), and its variance was calculated using Eq. (3) [13].

1 $$INB=\Delta E\left(K-ICER\right)$$

2 $$INB=\left(K\times \Delta E\right)-\Delta \mathrm{C}$$

3 $$Var\left(INB\right)={K}^{2}{\sigma}_{\Delta E}^{2}+{\sigma}_{\Delta C}^{2}-2K{\rho}_{\Delta C\Delta E}{\sigma}_{\Delta E}{\sigma}_{\Delta C}$$

where K is the WTP threshold, ΔE and ΔC are the incremental effectiveness and cost, σ is the standard deviation, and ${\rho}_{\Delta C\Delta E}$ is the correlation coefficient between ΔE and ΔC. A positive INB indicates cost-effectiveness at the specified WTP threshold, whereas a negative INB indicates that the intervention is not cost-effective at that threshold.

### Data preparation

Data preparation was conducted under two scenarios. Scenario 1 included studies reporting ΔC, ΔE, and 95% confidence intervals (*CI*) for ΔC and ΔE. Scenario 2 included studies that did not report measures of dispersion for ΔC and ΔE but provided cost-effectiveness PSA scatter plots. For studies in scenario 1, standard deviations were calculated from the reported 95% *CI* under the assumption of an approximately normal distribution. For studies in scenario 2, the minimum and maximum values of ΔC and ΔE were digitized from the PSA scatter plots, the number of PSA simulations served as the sample size parameter, and standard deviations were estimated using the method proposed by Wan et al. [25]. INB and variance were calculated accordingly, with specific methods detailed in Supplementary material 4. ΔC and ΔE were divided by the model population size to obtain per-person estimates. Study-reported WTP thresholds were used to calculate INB. When multiple thresholds were reported, the threshold common to all included studies from the same country was selected. If no WTP threshold was reported, the corresponding threshold was imputed from comparable included studies in that country. All cost estimates were converted to 2023 US dollars (USD) by first converting the local currency to USD using the exchange rate for the costing year, then adjusting for inflation using the gross domestic product (GDP) implicit price deflator, as recommended by Turner et al.’s adjustment method number 1 [26]. Inflation rates and currency conversion data were sourced from The World Bank. Details of currency conversion were provided in Supplementary material 4.

### Statistical analysis

Random-effects meta-analyses using the DerSimonian and Laird method were conducted separately for infants and older adults [27]. Separate univariable meta-regression analyses were performed for infants and older adults. Subgroup analyses were jointly stratified by target population and each of the following characteristics: geographic region, time horizon, funding source, and vaccine type. For each population-specific pooled analysis, one base-case estimate with an estimable variance was selected per study [28]. When multiple base-case estimates were reported within a study, the following hierarchical criteria were applied: (1) healthcare over societal perspective; (2) seasonal over year-round vaccination; (3) the target population most comparable with those in other studies; and (4) vaccine-specific estimates were retained separately when different vaccines were evaluated.

Heterogeneity was assessed using the *I*^2^ statistic and was considered only if the *I*^2^ value was > 25%. A covariate was considered a potential source of heterogeneity if *I*^2^ decreased by 50% or more. Publication bias was assessed using Egger's test and funnel plots [29]. If asymmetry was detected, contour-enhanced funnel plots were used to explore possible explanations. Leave-one-out sensitivity analyses were performed to assess the robustness of the pooled INB estimates. Analyses were conducted using Microsoft Excel 2021 (Microsoft Corporation, Redmond, United States) and R version 4.3.3 (R Foundation for Statistical Computing, Vienna, Austria), with a *P* value ≤ 0.05 considered statistically significant.

## Results

### Study selection

**Fig. 1:**
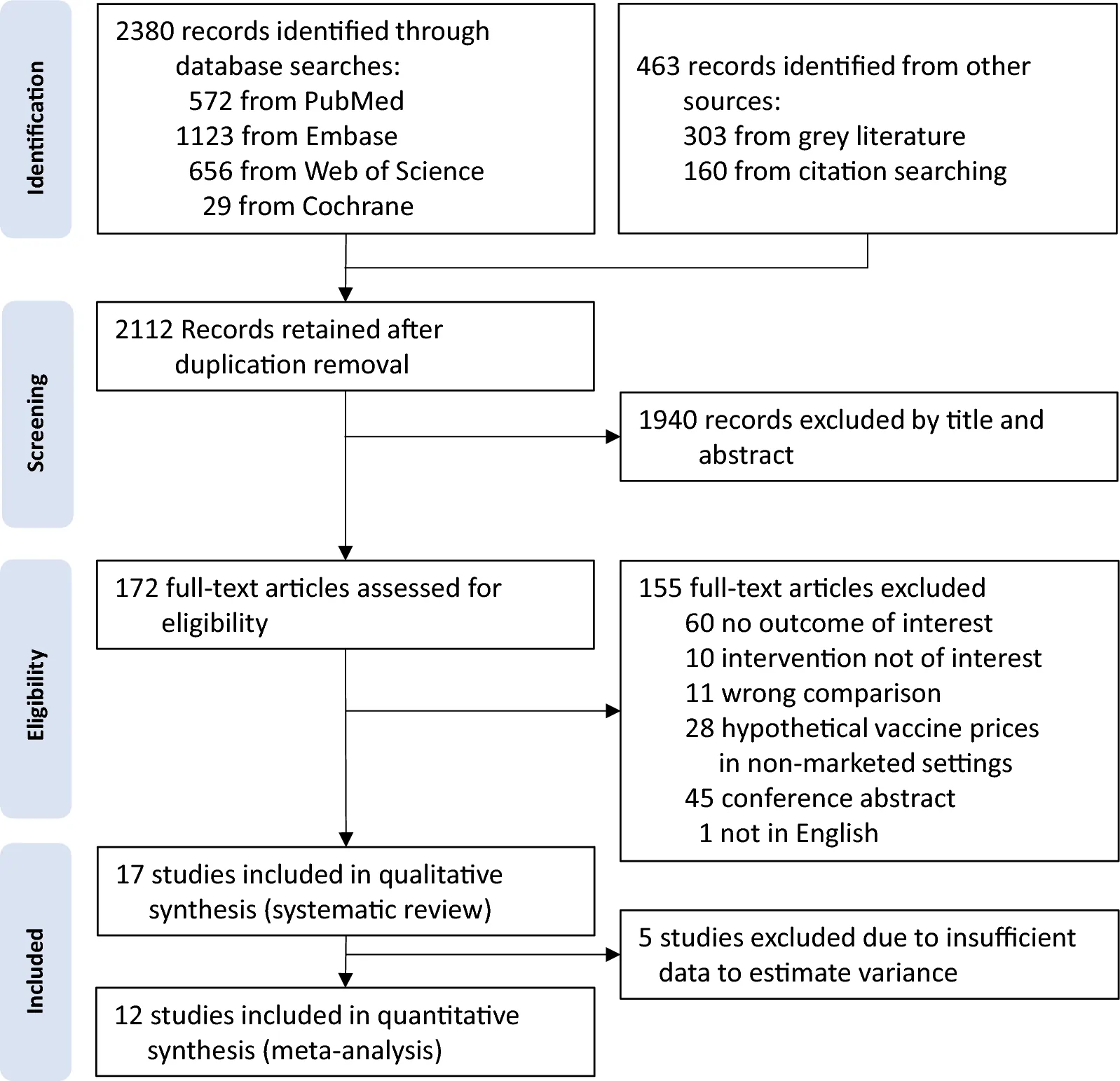
Flow diagram of study selection

A total of 2112 unique records were screened by title and abstract. After screening and full-text assessment, 17 studies were included in the systematic review. Of these, 12 contributed to quantitative synthesis: 11 to the primary meta-analysis and 1 to sensitivity analysis only (Fig. 1). One included study was a grey literature report. Selected studies excluded after full-text assessment and the reasons for exclusion were listed in Supplementary material 5 (Table S1).

### Study characteristics

A total of seventeen studies were included in the systematic review, and their characteristics are summarized in Table 1. All seventeen studies compared RSV vaccination with no vaccination [30–46] and were conducted in high-income settings where RSV vaccines were marketed. Six studies (35%) were conducted in the United States [30–32, 39, 40, 46], two studies (12%) in Canada [33, 34], eight studies (47%) in European countries [35–38, 41–44], and one study (6%) in China (Hong Kong) [45]. Adult vaccination was evaluated in nine studies (53%) [30–38], whereas maternal vaccination to protect infants was evaluated in eight studies (47%) [39–46]. Among adult-focused studies, three studies (18%) used lifetime horizons [35, 37, 38], three studies (18%) used 5-year horizons [30, 32, 36], and three studies (18%) used 2- or 3-year horizons [31, 33, 34]. Among infant-focused studies, seven studies (41%) used time horizons of no more than 1 year [39–42, 44–46], whereas one study (6%) used a 5-year horizon [43].

**Table 1:** Characteristics of the 17 included economic evaluations of RSV vaccination compared with no vaccination

Author (publication year)	Funding source	Geographic region	Target population	Modeling approach	Perspective	Discount rate (%)	Intervention	Price per dose (USD, 2023)	Vaccination timing	Coverage rate (%)	Time horizon	WTP (per QALY)	Currency year	Outcome measure
La et al. (2024) [30]	GSK	United States	Adults aged ≥ 60 years	Markov	Societal; healthcare sector	3.0	Arexvy	286.97	Seasonal	46.2–69.7	5 years	USD 50,000/100,000/150,000	2024	QALY
Hutton et al. (2024) [31]	US CDC	United States	Adults aged ≥ 60 years; vaccination thresholds of ≥ 60, ≥ 65, ≥ 70, ≥ 75, and ≥ 80 years	Decision analytic	Societal	3.0	Arexvy; Abrysvo	Arexvy: 290.15; Abrysvo: 305.70	Seasonal	20.0	2 years	USD 100,000/150,000	2022	QALY
Singer et al. (2025) [32]	GSK	United States	Adults aged 50–59 years at increased risk	Static multicohort Markov model	Societal; healthcare sector	3.0	Arexvy	286.97	Not specified	46.2	5 years	USD 50,000/100,000/150,000	2024	QALY
Rudd et al. (2025) [33]	None	Canada	Adults aged ≥ 50 years, stratified by age and chronic medical condition status	Static cohort cost-utility models	Health system	1.5	Arexvy; Abrysvo	Arexvy: 170.38; Abrysvo: 170.38	Seasonal	59.9	2 or 3 years	CAD 50,000	2023	QALY
Tuite et al. (2024) [34]	PHAC	Canada	Adults aged ≥ 50 years, stratified by age and chronic medical condition status	Static individual-based model	Health-system	1.5	Arexvy; Abrysvo	Arexvy: 170.38; Abrysvo: 170.38	Seasonal	58.6–83.4	3 years	CAD 50,000	2023	QALY
Szilcz et al. (2025) [35]	Pfizer	Sweden	High-risk adults aged 60–74 years and all adults aged 75–99 years	Markov	Societal; healthcare	3.0	Abrysvo	180.11	Seasonal	75.0	Lifetime	SEK 300,000	2024	QALY
Waize et al. (2025) [36]	GSK	Germany	Adults aged 50–59 years at increased risk and adults aged ≥ 60 years	Markov	Societal	3.0	Arexvy	225.68	Seasonal	75.0	5 years	EUR 50,000	2024	QALY
Gourzoulidis et al. (2024) [37]	Pfizer	Greece	Adults aged ≥ 60 years	Decision-analytic model with a Markov structure	Public payer	3.5	Abrysvo	217.62	Not specified	20.0–60.0	Lifetime	EUR 44,000	2024	QALY
Averin et al. (2025) [38]	Pfizer	Germany	Adults aged 60–99 years	Markov	Societal; healthcare system	3.0	Abrysvo	230.97	Seasonal	27.0–54.0	Lifetime	EUR 12,300–50,000	2023	QALY
Averin et al. (2025) [39]	Pfizer	United States	Pregnant women (protecting infants)	Markov	Societal	3.0	Abrysvo	295.00	Seasonal	54.9	1 year	USD 50,000/100,000/150,000	2023	QALY
Nguyen et al. (2025) [40]	US CDC	United States	Pregnant women (protecting infants)	Markov	Societal	3.0	Abrysvo	265.50	Seasonal	58.4	6 months	USD 150,000	2024	QALY
Álvarez Aldean et al. (2024) [41]	Pfizer	Spain	Pregnant women (protecting infants)	Markov	Societal; Spanish NHS	3.0	Abrysvo	180.03	Year-round	70.0	1 year	EUR 25,000	2023	QALY
Gourzoulidis et al. (2025) [42]	Pfizer	Greece	Pregnant women (protecting infants)	Markov	Public payer	3.5	Abrysvo	217.62	Year-round	19.5	1 year	EUR 44,000	2024	QALY
Li et al. (2025) [43]	KCE; University of Antwerp	Belgium	Pregnant women (protecting infants)	Static cohort model	Health care payer	3.0	Abrysvo	196.52	Seasonal	40.0	5 years	EUR 35,000	2024	QALY
Værnø et al. (2025) [44]	Pfizer	Norway	Pregnant women (protecting infants)	Markov	Societal; extended healthcare sector	0.0	Abrysvo	181.46	Seasonal	73.8	1 year	NOK 500,000	2024	QALY
Wang et al. (2025) [45]	None	China (Hong Kong)	Pregnant women (protecting infants)	Decision-analytic cohort model	Societal	3.0	Abrysvo	227.43	Year-round	100.0	1 year	USD 54,101	2024	QALY
Hutton et al. (2024) [46]	US CDC	United States	Pregnant women (protecting infants)	Decision analytic	Societal; health system	3.0	Abrysvo	305.70	Seasonal	50.0	1 year	Not reported	2022	QALY

WTP values are expressed in the price years reported in the “Currency year” column. CAD: Canadian dollar; EUR: euro; GSK: GlaxoSmithKline; KCE: Belgian Health Care Knowledge Centre; LY: life-year; NHS: National Health Service; NOK: Norwegian krone; PHAC: Public Health Agency of Canada; QALY: quality-adjusted life-year; RSV: respiratory syncytial virus; SEK: Swedish krona; US CDC: United States Centers for Disease Control and Prevention; WTP: willingness-to-pay

All included studies were model-based economic evaluations. Markov models were the most common framework, used in ten studies (59%) [30, 32, 35, 36, 38–42, 44], followed by decision-analytic models in four studies (24%) [31, 37, 45, 46], static cohort models in two studies (12%) [33, 43], and a static individual-based model in one study (6%) [34]. No included study used a dynamic transmission model. Abrysvo was evaluated in fourteen studies (82%) [31, 33–35, 37–46] and Arexvy in six studies (35%) [30–34, 36], with three studies (18%) evaluating both vaccines [31, 33, 34]. Vaccine prices ranged from USD 170.38 to USD 305.70 per dose (2023 USD), with the highest prices reported in the United States [30–32, 39, 40, 46] and the lowest in Canada [33, 34]. Seasonal vaccination was evaluated in thirteen studies (76%) [30, 31, 33–36, 38–40, 43–46], while five studies (29%) assessed year-round vaccination [39, 41–43, 45]. Three studies (18%) evaluated both seasonal and year-round vaccination [39, 43, 45], and vaccination timing was not specified in two studies (12%) [32, 37].

The societal perspective was reported in twelve studies (71%) [30–32, 35, 36, 38–41, 44–46], while healthcare, payer, or health-system perspectives were reported in twelve studies (71%) [30, 32–35, 37, 38, 41–44, 46]. Seven studies (41%) reported both societal and healthcare, payer, or health-system perspectives [30, 32, 35, 38, 41, 44, 46]. Ten studies (59%) were funded by vaccine manufacturers [30, 32, 35–39, 41, 42, 44], including seven studies (41%) funded by Pfizer [35, 37–39, 41, 42, 44] and three studies (18%) funded by GSK [30, 32, 36]. The remaining seven studies (41%) reported public or other non-industry funding, or no external funding [31, 33, 34, 40, 43, 45, 46].

### Risk of bias assessment

Most studies (16/17, 94%) were assessed as having no apparent inefficient comparator bias or bias related to treatment effects. All studies were assessed as having no apparent cost measurement/omission bias or wrong model bias. Bias related to narrow perspective, sponsor, structural assumptions, limited time horizon, and limited sensitivity analysis varied across studies. Based on the three prespecified key items—limited sensitivity analysis bias, wrong model bias, and bias related to treatment effects—twelve studies (71%) were classified as low risk of bias, four studies (24%) as moderate risk of bias, and one study (6%) as high risk of bias (Supplementary material 6).

### Primary meta-analysis

The primary pooled analyses included one estimate per study, selected using the prespecified hierarchy. Accordingly, the healthcare-perspective estimate was selected for Værnø et al. [44], seasonal vaccination estimates for Averin et al. [39], Li et al. [43], and Wang et al. [45], and the estimate for adults aged ≥ 60 years was selected for Hutton et al. [31]. The Abrysvo and Arexvy estimates from Hutton et al. [31] were analyzed in separate parallel analyses, with only one vaccine-specific estimate included in each pooled analysis. Singer et al. [32] evaluated adults aged 50–59 years with chronic obstructive pulmonary disease and increased risk of severe RSV disease. This study was excluded from the primary pooled analysis because of limited population comparability and included in sensitivity analysis.

### Synthesis of results

**Fig. 2:**
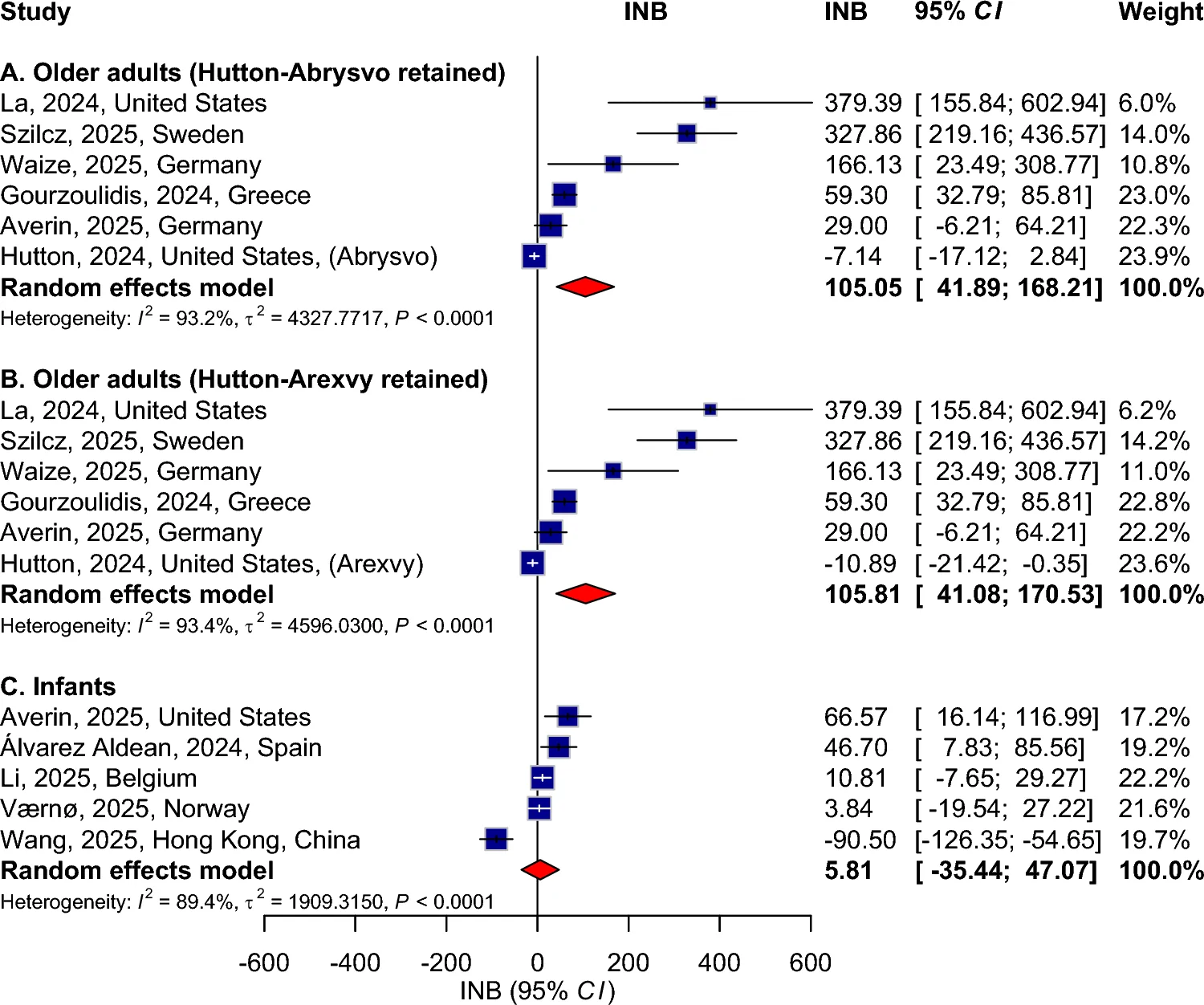
Population-specific forest plots of the incremental net benefit of RSV vaccination. Panel **A** shows the older-adult analysis retaining the Hutton-Abrysvo estimate, panel **B** shows the older-adult analysis retaining the Hutton-Arexvy estimate, and panel **C** shows the infant analysis. *CI*: confidence interval; INB: incremental net benefit

Among older adults, the pooled INBs were USD 105.05 (95% *CI:* 41.89–168.21; *I*^2^ = 93.2%) with Hutton-Abrysvo retained and USD 105.81 (95% *CI:* 41.08–170.53; *I*^2^ = 93.4%) with Hutton-Arexvy retained. Among infants, the pooled INB was USD 5.81 (95% *CI:* − 35.44–47.07; *I*^2^ = 89.4%) (Fig. 2). At the study-specific WTP thresholds, these findings suggested that RSV vaccination might be cost-effective among older adults in post-marketing settings, whereas the cost-effectiveness of maternal vaccination remained uncertain. These estimates should be interpreted cautiously given the substantial between-study heterogeneity. Egger's tests did not indicate funnel-plot asymmetry for infants (*P* = 0.953) but indicated asymmetry in both older-adult scenarios (*P* = 0.014 and *P* = 0.015) (Figure S1 and Table S2 in Supplementary material 7). Contour-enhanced funnel plots suggested that heterogeneity may have contributed to the asymmetry, although publication bias or other small-study effects could not be excluded (Figure S2 in Supplementary material 7).

Sensitivity analyses showed that, for older adults, pooled INBs remained positive and statistically significant in all leave-one-out analyses (Tables S4–S5 in Supplementary material 8). Including Singer et al. had minimal impact on the pooled estimates, with their direction and statistical significance unchanged (Figure S3 in Supplementary material 8) [32]. Among infants, excluding Averin et al. [39] or Álvarez Aldean et al. [41] changed the pooled INBs from positive to negative, but neither estimate was statistically significant. In contrast, excluding Wang et al. [45] yielded a positive and statistically significant pooled INB (USD 23.98; 95% *CI:* 0.45–47.51), with* I*^2^ decreasing from 89.4% to 60.5% (Table S3 in Supplementary material 8).

Univariable meta-regression showed a statistically significant association between geographic region and INB among infant studies, with *I*^2^ decreasing from 89.4% to 43.4% after including geographic region as a covariate. This suggested that geographic region may partly explain the observed heterogeneity. No statistically significant associations with INB were observed for vaccine price, WTP threshold, funding source, or time horizon (Table S6 in Supplementary material 9). Among older-adult studies, geographic region, WTP threshold, funding source, and time horizon were not significantly associated with INB. The association between vaccine type and INB was statistically significant when the Hutton-Abrysvo estimate was retained (*P* = 0.045), but not when the Hutton-Arexvy estimate was retained (*P* = 0.970). Residual heterogeneity remained high across the meta-regression models for older adults (Tables S7–S8 in Supplementary material 9). Given the limited number of studies within each population, the meta-regression findings were considered exploratory.

### Subgroup analyses

**Fig. 3:**
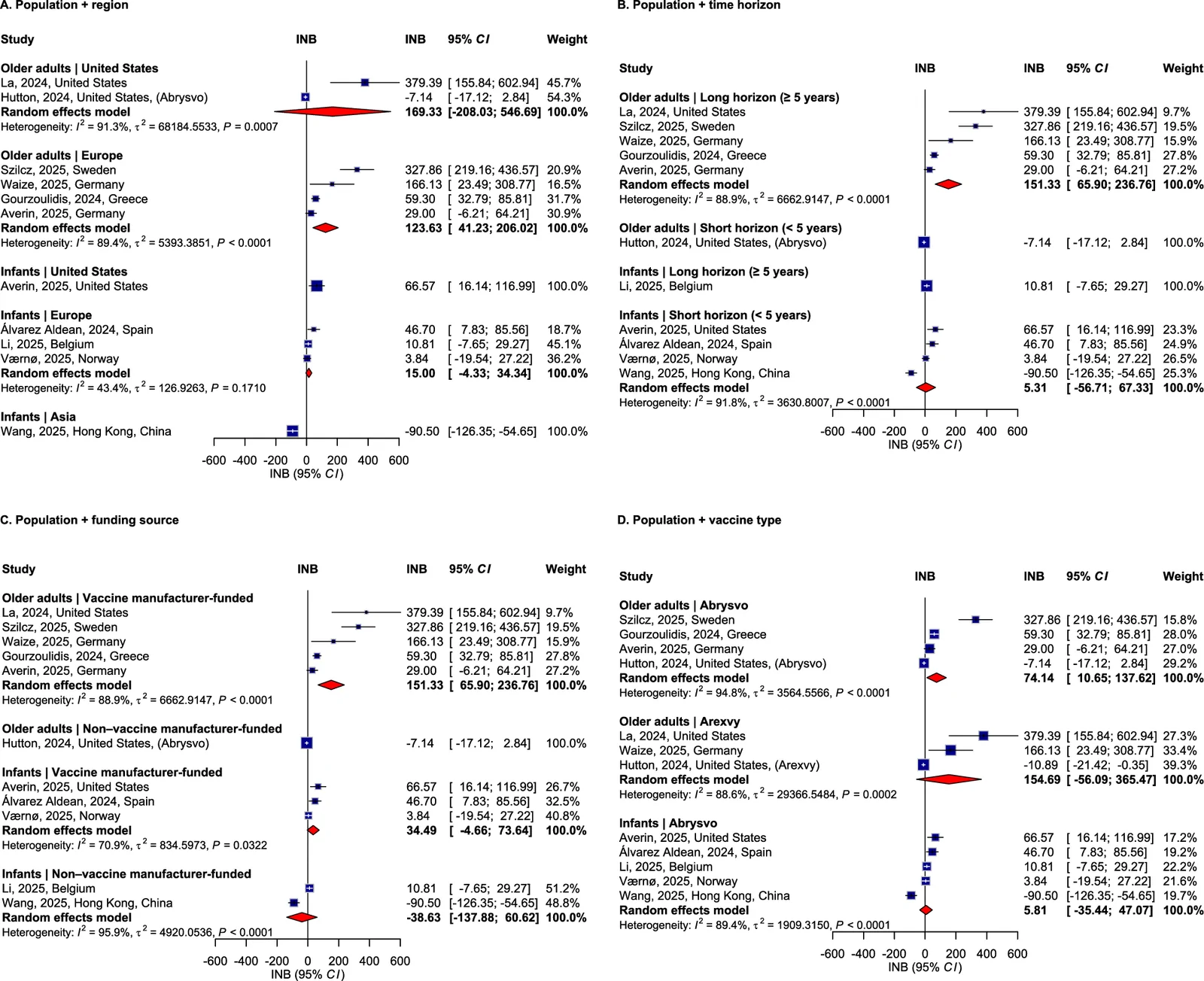
Population-specific subgroup analyses of incremental net benefit for RSV vaccination. Subgroup analyses stratified by target population and (**A**) region, (**B**) time horizon, (**C**) funding source, and (**D**) vaccine type. Panels **A**–**C** retained the Abrysvo estimate from Hutton et al. [31] to avoid double-counting correlated estimates from the same study. Alternative analyses using the Arexvy estimate from Hutton et al. were presented in Supplementary material 8 (Figure S4). *CI*: confidence interval; INB: incremental net benefit

Population-specific subgroup analyses showed variation by geographic region, time horizon, funding source, and vaccine type (Fig. 3). In the regional analysis, the pooled INBs among older adults were USD 123.63 (95% *CI:* 41.23–206.02; *I*^2^ = 89.4%) in Europe and USD 169.33 (95% *CI:* − 208.03–546.69; *I*^2^ = 91.3%) in the United States. Both estimates were positive, but statistical significance was observed only in Europe. Among infants, the pooled INB in Europe was USD 15.00 (95% *CI:* − 4.33–34.34; *I*^2^ = 43.4%), with no statistically significant evidence. Between-study heterogeneity was lower in the European infant subgroup than in the overall infant analysis (*I*^2^ = 43.4% vs 89.4%), suggesting that geographic region may partly contribute to the observed heterogeneity (Fig. 3A).

In the time-horizon analysis, the pooled INB among older adults in studies with long time horizons (≥ 5 years) was USD 151.33 (95% *CI:* 65.90–236.76; *I*^2^ = 88.9%), which was positive and statistically significant. Among infants in studies with short time horizons (< 5 years), the pooled INB was USD 5.31 (95% *CI:* − 56.71–67.33; *I*^2^ = 91.8%) and was not statistically significant. Only one study contributed to each of the two subgroups: older adults with short time horizons and infants with long time horizon (Fig. 3B).

In the analysis by funding source, the pooled INB among older adults in studies funded by vaccine manufacturers was USD 151.33 (95% *CI:* 65.90–236.76; *I*^2^ = 88.9%), which was positive and statistically significant. Among infants, manufacturer-funded studies had a pooled INB of USD 34.49 (95% *CI:* − 4.66–73.64; *I*^2^ = 70.9%), whereas studies without manufacturer funding had a pooled INB of USD − 38.63 (95% *CI:* − 137.88–60.62; *I*^2^ = 95.9%). Neither estimate was statistically significant. Only one study was included in the subgroup of older adults without manufacturer funding (Fig. 3C).

In the analysis by vaccine type, the pooled INBs among older adults were USD 74.14 (95% *CI:* 10.65–137.62; *I*^2^ = 94.8%) for Abrysvo and USD 154.69 (95% *CI:* − 56.09–365.47; *I*^2^ = 88.6%) for Arexvy. Both estimates were positive, but only the Abrysvo estimate was statistically significant. Among infants, all studies evaluated Abrysvo, with a pooled INB of USD 5.81 (95% *CI:* − 35.44–47.07; *I*^2^ = 89.4%) (Fig. 3D).

Replacing the Hutton-Abrysvo estimate with the Hutton-Arexvy estimate produced minor changes in the subgroup estimates, without changing their direction or statistical significance (Figure S4 in Supplementary material 8). Because several subgroups contained a small number of studies, the analyses had limited statistical power and should be interpreted cautiously.

### Age- and risk-based targeting in older adults

**Fig. 4:**
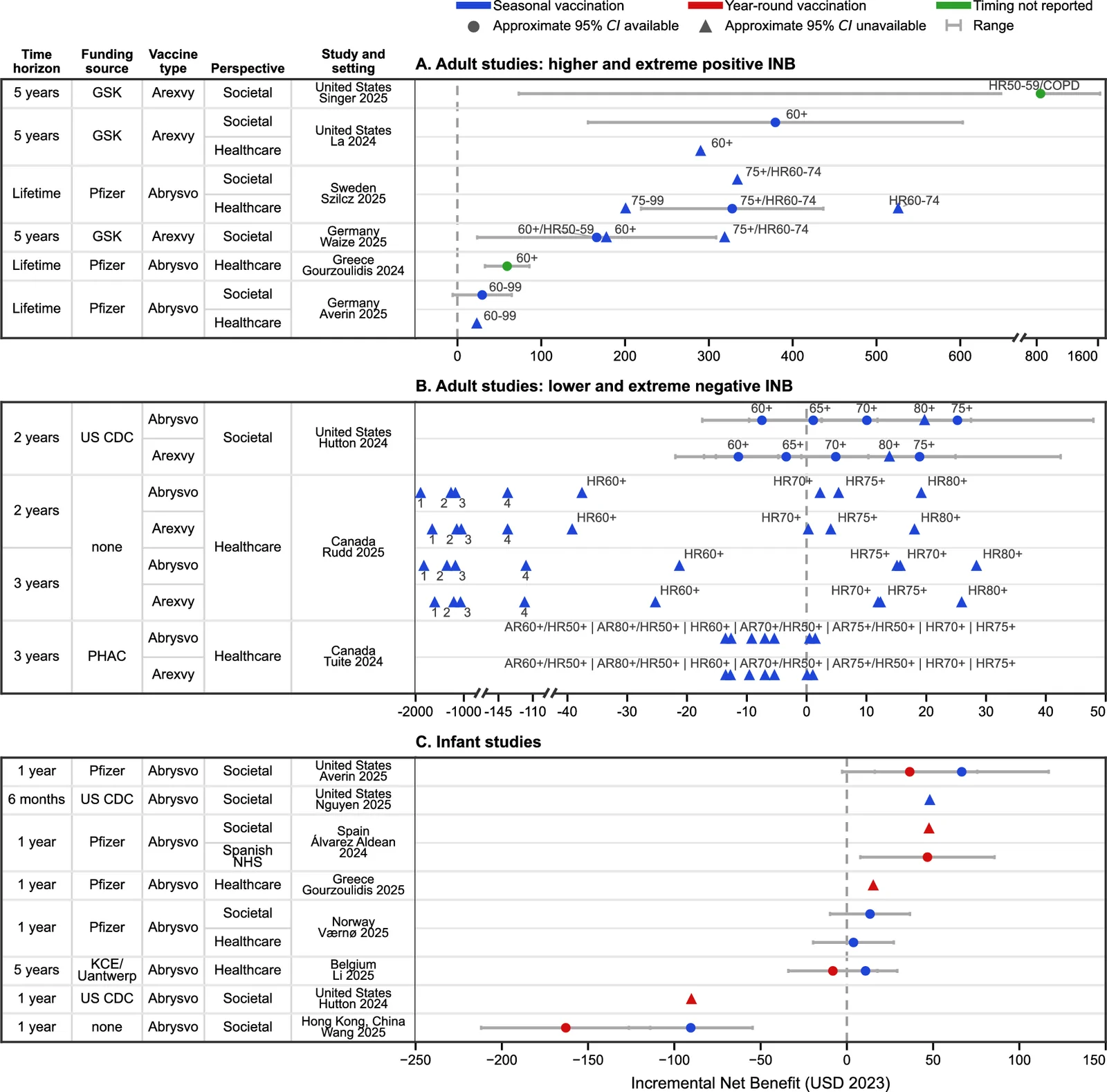
Incremental net benefit of RSV vaccination by adult target population and maternal vaccination timing. Points within each adult study row represent different vaccination target populations, whereas those within each infant study row represent different vaccination timing strategies. AR75+/HR50 + represents average-risk adults aged 75 years and older and high-risk adults aged 50 years and older. In Rudd et al. [33], labels 1–4 represent AR60+/HR50+, AR70+/HR50+, AR75+/HR50+, and AR80+/HR50+, respectively. INB estimates for the PHAC model in Rudd et al. [33] were calculated from digitized incremental cost and quality-adjusted life-year (QALY) data using WebPlotDigitizer version 4.4 [23]. *AR*: average risk; *HR*: high risk; *COPD*: chronic obstructive pulmonary disease; *GSK*: GlaxoSmithKline; *KCE*: Belgian Health Care Knowledge Centre; *NHS*: National Health Service; *PHAC*: Public Health Agency of Canada; *QALY*: quality-adjusted life-year; *UAntwerp*: University of Antwerp; *US CDC*: United States Centers for Disease Control and Prevention﻿

Among older adults, INB estimates varied across age- and risk-based vaccination strategies (Fig. 4A, B). In Hutton et al. [31], INB estimates increased as the minimum vaccination age rose from 60 to 75 years, but declined slightly at 80 years. In Rudd et al. [33], broader vaccination strategies targeting both average-risk and high-risk adults yielded markedly negative INBs, whereas strategies targeting high-risk adults at older ages produced more favorable estimates. Tuite et al. [34] similarly favored vaccination strategies targeting high-risk adults aged 70 years and older over broader age-based vaccination. Across these studies, vaccination strategies targeting older age groups or selected high-risk populations yielded higher INB estimates than broader vaccination strategies, although the most favorable age and risk criteria differed across studies.

### Funding source and time horizon in older adults

Among adult studies, manufacturer-funded evaluations used time horizons of at least 5 years or lifetime horizon and reported higher INB estimates (Fig. 4A). In contrast, studies without manufacturer funding used 2- or 3-year horizons and reported lower INB estimates (Fig. 4B). Because funding source and time horizon were closely related, their independent associations with INB could not be determined.

### Timing of maternal vaccination

In three studies directly comparing seasonal and year-round maternal vaccination, seasonal strategies yielded higher INBs [39, 43, 45] (Fig. 4C). Averin et al. [39] found that broader vaccination windows prevented more RSV cases but were less economically favorable than the narrower base-case strategy. Nguyen et al. [40] reported that maternal vaccination was cost-saving for infants born in October–December, cost-effective for those born in January, and not cost-effective for those born in February. These descriptive findings suggested that targeting vaccination to populations and periods with greater expected RSV burden may improve its economic value.

## Discussion

This systematic review and meta-analysis synthesized evidence on the cost-effectiveness of RSV vaccination in post-marketing settings, with separate analyses for older adults and infants. Among older adults, the pooled INB was positive and statistically significant at the study-specific WTP thresholds and remained so in sensitivity analyses. For maternal vaccination, the pooled INB was not statistically significant and was sensitive to study exclusion. However, substantial between-study heterogeneity warrants cautious interpretation of these findings.

After stratification by target population and vaccine type, the pooled INB for Abrysvo among older adults was positive and statistically significant. The pooled INBs for Arexvy among older adults and Abrysvo for infants were also positive, but neither reached statistical significance. In addition to maternal vaccination, long-acting monoclonal antibodies such as nirsevimab have demonstrated protection against RSV-related lower respiratory tract disease and hospitalization in infants [47]. As this review focused on RSV vaccines, monoclonal antibody strategies were not included. Future systematic reviews and meta-analyses could compare the cost-effectiveness of maternal vaccination and infant monoclonal antibody prophylaxis.

After stratification by population and time horizon, the pooled INB among older adults in studies with horizons of at least 5 years was positive and statistically significant. Among infants, the pooled INB in studies with horizons of less than 5 years was positive but not statistically significant. The other two subgroups each contained only one study. Longer time horizons may capture additional health gains and downstream cost offsets, particularly when vaccination prevents severe disease or mortality. The influence of time horizon may differ between populations because maternal vaccination provides passive protection through transferred antibodies that wane during infancy, whereas vaccination in older adults induces active immunity [48, 49].

Regional differences in pooled INB estimates were observed after stratification by target population and geographic region. Among older adults, the European pooled INB was positive and statistically significant. The United States pooled estimate was not statistically significant and was highly imprecise, as only La et al. and one Hutton scenario contributed to this stratum [30, 31]. Among infants, the European pooled INB was not statistically significant, while the United States and Asian subgroups each contained only one study. At a WTP threshold of USD 150,000 per QALY, Hutton et al. found that neither Abrysvo nor Arexvy was cost-effective among adults aged 60 years and older, whereas La et al. found Arexvy to be cost-effective in the same age group [30, 31]. These differences may partly reflect differences in model assumptions, including a lower assumed incidence of RSV-related hospitalization and a shorter assumed duration of vaccine protection in the Hutton model than in the La model [30, 31]. Hutton et al. also did not account for effects on non–medically attended RSV illness, downstream sequelae, or out-of-hospital RSV-associated deaths [31]. The low INB in the China (Hong Kong) study may partly reflect lower RSV-related healthcare costs than those assumed in a US maternal vaccination model, resulting in smaller cost offsets from vaccination [45, 46].

Economic findings also varied by funding source. Among older adults, the pooled INB was positive and statistically significant in manufacturer-funded studies. Among infants, neither funding subgroup showed a statistically significant pooled INB. Manufacturer-funded studies of older adults used time horizons of at least 5 years, whereas the only study without manufacturer funding used a shorter horizon. The observed differences may therefore partly reflect variations in time horizon rather than funding source alone. Funding source should be considered alongside time horizon, model assumptions, and included benefit components, rather than used as the sole criterion for excluding studies.

The descriptive analyses showed that INB estimates varied across vaccination target populations and timing strategies. Among adults, strategies targeting selected older age groups or those at increased risk of severe RSV disease yielded higher INB estimates than broader age-based vaccination strategies [31, 33, 34]. Other economic evaluations also found that the cost-effectiveness of RSV vaccination varied with the age and risk profile of the target population [50, 51]. In the three studies that directly compared seasonal and year-round maternal vaccination, seasonal vaccination consistently yielded higher INBs [39, 43, 45]. These findings suggested that tailoring vaccination eligibility and timing may improve the economic value of RSV vaccination programs. Although the included evidence was limited to high-income settings, prioritizing vaccination target groups and optimizing vaccination timing are also important considerations when developing vaccination strategies in resource-constrained settings.

The choice of an appropriate summary measure is important for the quantitative synthesis of economic evaluations. Pooling ICERs is generally inappropriate because these ratios are highly sensitive to small changes in incremental health gains and can produce extreme or ambiguous values when incremental effects approach zero or become negative [52]. We therefore used INB as the primary outcome measure. Its linear form provides a more interpretable basis for comparing and pooling economic evaluation results at specified WTP thresholds [13]. Expressing results on a common monetary scale facilitates quantitative synthesis across studies, although interpretation remains dependent on study-specific thresholds and modeling assumptions [14–20]. In addition, restricting the review to evaluations using locally established vaccine prices from official or recognized sources reduced reliance on hypothetical pricing assumptions and maximum cost-effective price-per-dose estimates, improving the transparency and interpretability of the findings.

Several limitations should be acknowledged. First, substantial between-study heterogeneity limits the interpretation and generalizability of the pooled INB estimates. Although subgroup analyses and univariable meta-regression identified geographic region as a potential source of heterogeneity among infant studies, the limited number of studies and correlations among study characteristics made it difficult to assess its independent contribution. Substantial residual heterogeneity also remained among older-adult studies. Second, all included economic evaluations were conducted in high-income settings, limiting generalizability of the findings to low- and middle-income countries, where RSV burden, healthcare costs, affordability, and implementation conditions may differ. Third, the review focused on RSV vaccines and excluded long-acting monoclonal antibodies such as nirsevimab. Therefore, the infant findings should be interpreted in the context of maternal vaccination rather than all infant RSV prevention strategies. Finally, the lack of prospective registration of the review protocol may increase the risk of selective reporting and post hoc methodological decisions [53]. Nevertheless, a protocol was developed before study selection and is provided in Supplementary material 1, together with any protocol deviations, to support transparency and reproducibility.

## Conclusions

This study provided a quantitative synthesis of the cost-effectiveness of RSV vaccines in post-marketing settings using the INB framework. RSV vaccination might be cost-effective for older adults in post-marketing settings at the study-specific WTP thresholds, whereas the evidence for infants remained inconclusive. Economic outcomes varied across populations, settings, and analytic assumptions. Descriptive findings further suggested that age- and risk-based targeting in older adults and seasonal maternal vaccination may yield more favorable INB estimates. Given the substantial between-study heterogeneity and the small number of studies in several subgroups, the pooled and subgroup estimates should be interpreted cautiously in light of local RSV epidemiology, healthcare costs, vaccination policy design, and implementation contexts.

## Supplementary Information

Supplementary Material 1. Review protocol and protocol deviations. Review question, eligibility criteria, planned review methods, and documented protocol deviations

Supplementary Material 2. PRISMA 2020 checklist. Completed PRISMA 2020 checklist identifying where the corresponding information is reported in the main manuscript and supplementary files

Supplementary Material 3. Search strategy. Database-specific search strategies and grey literature search methods

Supplementary Material 4. Scenario for data preparation. Equations and assumptions for calculating incremental net benefit and its variance, estimating missing dispersion measures, and converting monetary values to 2023 US dollars

Supplementary Material 5. Studies excluded after full-text assessment. Selected studies and related reports excluded after full-text assessment, with reasons for exclusion (Table S1)

Supplementary Material 6. Assessment of risk of bias. Item-level and overall risk-of-bias assessments of the 17 included economic evaluations using the modified Economic Evaluation Bias checklist

Supplementary Material 7. Assessment of publication bias. Population-specific funnel plots, Egger regression tests, and contour-enhanced funnel plots (Figures S1–S2 and Table S2)

Supplementary Material 8. Sensitivity analyses. Leave-one-out analyses, sensitivity analyses including Singer et al., and alternative vaccine-scenario subgroup analyses (Tables S3–S5 and Figures S3–S4)

Supplementary Material 9. Meta-regression. Description of data: Population-specific univariable meta-regression results for infants and older adults (Tables S6–S8)

## Data Availability

The datasets generated and/or analysed during the current study are available from the corresponding author on reasonable request.

## Abbreviations

ACIP: Advisory Committee on Immunization Practices
AR: Average risk
CAD: Canadian dollar
CDA-AMC: Canada’s Drug Agency
CDC: Centers for Disease Control and Prevention
*CI*: Confidence interval
COPD: Chronic obstructive pulmonary disease
ECOBIAS: Economic Evaluation Bias
EUR: Euro
GDP: Gross domestic product
GSK: GlaxoSmithKline
HR: High risk
HTA: Health technology assessment
ICER: Incremental cost-effectiveness ratio
INAHTA: International Network of Agencies for Health Technology Assessment
INB: Incremental net benefit
IRIS: Institutional Repository for Information Sharing
ISPOR: International Society for Pharmacoeconomics and Outcomes Research
KCE: Belgian Health Care Knowledge Centre
LY: Life-year
NHS: National Health Service
NOK: Norwegian krone
PHAC: Public Health Agency of Canada
PRISMA: Preferred Reporting Items for Systematic Reviews and Meta-Analyses
PSA: Probabilistic sensitivity analysis
QALY: Quality-adjusted life-year
RSV: Respiratory syncytial virus
RSVPreF: Bivalent RSV prefusion F vaccine
RSVPreF3 OA: RSV prefusion F protein vaccine for older adults
SEK: Swedish krona
US: United States
US CDC: United States Centers for Disease Control and Prevention
USD: United States dollar
WHO: World Health Organization
WTP: Willingness-to-pay
ΔC: Incremental costs
ΔE: Incremental effects
