# Supplementary material for: Cost-effectiveness of respiratory syncytial virus vaccines in post-marketing settings: a systematic review and meta-analysis
Source: Infect Dis Poverty. 2026 Nov 14;15:117. doi: 10.1186/s40249-026-01520-y (PMC13770325; doi:10.1186/s40249-026-01520-y)
Supplement: Supplementary file 1 [file 40249_2026_1520_MOESM1_ESM.docx]

# Supplementary material 1. Review protocol and protocol deviations

**Review title**

Cost-effectiveness of respiratory syncytial virus vaccines in post-marketing settings: a systematic review and meta-analysis

**Review question**

What is the cost-effectiveness of marketed respiratory syncytial virus (RSV) vaccines compared with no vaccination in settings where RSV vaccines are commercially available?

The review aims to synthesize evidence from published economic evaluations and to estimate the pooled incremental net benefit (INB) of RSV vaccination compared with no vaccination.

**Background and rationale**

RSV is a major cause of respiratory illness, hospitalization, and mortality, especially among infants and older adults. Several RSV vaccines have recently entered clinical use, including vaccines targeting older adults and maternal immunization to protect infants. With the increasing availability of RSV vaccines, economic evidence is needed to inform immunization policy and resource allocation.

Previous systematic reviews have summarized the cost-effectiveness of RSV prevention strategies descriptively, but quantitative synthesis of RSV vaccine cost-effectiveness evidence remains limited. Because direct pooling of incremental cost-effectiveness ratios (ICERs) is methodologically problematic, this review uses INB as a common linear outcome for quantitative synthesis.

**Population**

No restriction will be placed on population. No restriction will be placed on country or region, but included evaluations must be conducted in settings where the RSV vaccine being evaluated was commercially available or had entered policy-relevant post-marketing use at the time of the evaluation.

**Intervention**

The intervention of interest is vaccination against RSV using marketed RSV vaccines, including but not limited to:

- Abrysvo [bivalent RSV prefusion F vaccine (RSVpreF)];
- Arexvy [RSV prefusion F protein vaccine for older adults (RSVPreF3 OA)];
- mRESVIA or other marketed RSV vaccines, if eligible economic evaluations are available.

**Comparator**

The comparator is no RSV vaccination or no intervention. Studies without a standalone vaccine-only arm or without a no-vaccination comparator will be excluded.

**Types of studies to be included**

Eligible studies will include full economic evaluations of RSV vaccination, including studies evaluating the cost-utility or cost-effectiveness of RSV vaccination.

**Exclusion criteria**

Studies will be excluded if they:

1. focus only on cost of illness or economic burden without evaluating vaccination;
2. evaluate hypothetical RSV vaccines or vaccines not marketed in the study setting at the time of evaluation;
3. do not compare RSV vaccination with no vaccination or standard care without vaccination;
4. do not provide sufficient information to extract or calculate cost-effectiveness outcomes;
5. are conference abstracts without sufficient full-text data;
6. are narrative reviews, systematic reviews, editorials, commentaries, or letters;
7. are not available in English.

**Search strategy**

Electronic database searches will be conducted in PubMed. The search will include terms related to RSV, vaccination, and economic evaluation. Two reviewers will independently screen titles and abstracts. Full texts of potentially eligible studies will then be assessed independently by the same two reviewers. Disagreements will be resolved through discussion, and unresolved disagreements will be adjudicated by a third reviewer.

**Data extraction**

Data will be extracted using a predefined extraction form. Two reviewers will independently extract data, with disagreements resolved through discussion or consultation with a third reviewer**.** The following information will be extracted:

- first author;
- publication year;
- title;
- country or region;
- target population;
- vaccine type;
- vaccination strategy and dose;
- comparator;
- coverage rate;
- vaccine price or cost per dose;
- model type;
- model population size;
- analytic perspective;
- time horizon;
- discount rate;
- currency and price year;
- funding source;
- outcome measure, such as quality-adjusted life-year (QALY), disability-adjusted life-year (DALY), or life-years;
- ICER;
- incremental cost, ΔC;
- incremental effect, ΔE;
- willingness-to-pay (WTP) threshold;
- uncertainty estimates, including standard error (SE), confidence interval (*CI*), or probabilistic sensitivity analysis (PSA) outputs;
- availability of PSA;
- data required to calculate INB and its variance.
- model population size

If multiple scenarios are reported, the base-case analysis will be prioritized. If more than one base-case analysis is reported, the analysis most consistent with the study’s stated preferred scenario will be used.

**Primary outcome**

The primary outcome is the INB of RSV vaccination compared with no vaccination.

INB will be calculated as:

$$INB=\Delta E\left( K-ICER \right)$$

$$INB=\left( K\times\Delta E \right)-\Delta C$$

$$Var\left( INB \right)=K^{2}\sigma_{\Delta E}^{2}+\sigma_{\Delta C}^{2}-2K\rho_{\Delta C\Delta E}\sigma_{\Delta E}\sigma_{\Delta C}$$

where K is the WTP threshold, ΔE is the incremental health effect, and ΔC is the incremental cost. $\sigma$ is the standard deviation (SD), and $\rho_{\Delta C\Delta E}$ is the correlation coefficient between ΔE and ΔC.

**Data preparation and standardization**

Where studies report ΔC, ΔE, and uncertainty measures, INB and its variance will be calculated directly.

Where studies report ICERs and 95% *CIs*, SDs will be derived under the assumption of approximate normality.

Where studies do not report numerical uncertainty measures but provide PSA scatter plots, PSA plots will be digitized using WebPlotDigitizer version 4.4. The minimum and maximum values of ΔC and ΔE will be extracted, and SDs will be estimated using the method proposed by Wan et al [1]. The correlation between ΔC and ΔE will be estimated from digitized PSA points where possible. ΔC and ΔE will be converted to per-person estimates by dividing by the model population size.

All cost estimates will be converted to 2023 United States dollars (USD). Local currency values will first be converted to USD using the exchange rate corresponding to the study’s costing year, then adjusted for inflation using gross domestic product (GDP) implicit price deflators. Exchange rates and inflation data will be obtained from the World Bank or other publicly available macroeconomic data sources.

**Risk-of-bias assessment**

Risk of bias will be assessed using the modified Economic Evaluation Bias (ECOBIAS) checklist. Each item will be rated as: yes, partly, unclear, no. Three key items will be used for the overall risk-of-bias classification:

1. limited sensitivity analysis bias;
2. incorrect model bias;
3. bias related to treatment effects.

Studies rated “yes” on all three key items will be classified as having low risk of bias. Studies with one or more “partly” or “unclear” ratings will be classified as moderate risk of bias. Studies with at least one “no” rating among these key items will be classified as high risk of bias.

Two reviewers will independently assess risk of bias, with disagreements resolved through discussion or consultation with a third reviewer.

**Data synthesis**

A structured descriptive synthesis will first be conducted to summarize study characteristics and study-specific cost-effectiveness findings. For studies with sufficient data, random-effects meta-analysis will be performed to pool INB estimates. The DerSimonian and Laird method will be used as the primary random-effects approach.

**Assessment of heterogeneity**

Statistical heterogeneity will be assessed using *I*² statistic. Heterogeneity will be considered notable if *I*² is greater than 25%.

**Planned subgroup analyses**

Predefined subgroup analyses will be conducted according to:

1. target population (older adults, maternal immunization / infants)
2. vaccine type (Abrysvo / RSVpreF, Arexvy / RSVPreF3 OA, other marketed RSV vaccines where available)
3. time horizon (shorter time horizon, defined as < 5 years, longer time horizon, defined as ≥ 5 years or lifetime)
4. geographic region
5. funding source (vaccine manufacturer funding, no vaccine manufacturer funding / public / non-industry funding)

**Meta-regression**

Univariable meta-regression analyses will be performed to explore associations between study-level covariates and INB. Potential covariates will include:

- target population;
- vaccine type;
- vaccine price;
- WTP threshold;
- time horizon;
- geographic region;
- funding source;

**Publication bias**

Publication bias will be assessed using funnel plots and Egger’s regression test, where the number of studies is sufficient.

Sensitivity analyses. Sensitivity analyses will be performed using the leave-one-out method, whereby one study is excluded at a time.

**Review team**

- M.M.: conceptualization, data extraction, formal analysis, visualization, manuscript drafting.
- Y.D**.**: conceptualization, data extraction, supervision, manuscript review.
- Z.D.: methodology, validation, adjudication of disagreements, manuscript review.
- Y.Z.: supervision, project administration, funding acquisition.

**Funding**

This work is supported by the National Natural Science Foundation of China and the Fundamental Research Plan of Shenzhen Government.

**Conflicts of interest**

The authors declare no competing interests.

**Protocol deviations**

| No. | Original protocol | Difference in the final review | Rationale |
| --- | --- | --- | --- |
| 1 | PubMed was the only planned bibliographic database. | Embase, Web of Science, and the Cochrane Library were added. | To improve search comprehensiveness and reduce the risk of missing eligible studies. |
| 2 | No formal grey literature search was planned. | Grey literature searches were added across International Network of Agencies for Health Technology Assessment (INAHTA), Google Scholar, Canada’s Drug Agency (CDA-AMC) reports, World Health Organization (WHO) Institutional Repository for Information Sharing (IRIS), and Centers for Disease Control and Prevention (CDC) Advisory Committee on Immunization Practices (ACIP) meeting slides. | To identify relevant reports and unpublished or non-peer-reviewed evidence. |
| 3 | Citation searching was not specified. | Citation searching of relevant reviews was added. | To identify additional studies that may have been missed through database searches. |
| 4 | Random-effects meta-analysis was planned for all studies with sufficient data; population-specific pooling was not prespecified. | Random-effects meta-analyses were conducted separately for infant and older-adult populations. | To avoid pooling clinically distinct target populations and to improve interpretability in the presence of between-population heterogeneity. |
| 5 | The protocol did not specify how multiple correlated vaccine scenarios from the same study would be handled. | For Hutton et al., one older-adult vaccine scenario was retained at a time, and the analysis was repeated using the alternative scenario. | To avoid double-counting correlated estimates from the same study while assessing the robustness of the pooled result to vaccine-scenario selection. |
| 6 | Subgroup analyses were planned separately by target population, vaccine type, time horizon, geographic region, and funding source. | Subgroup analyses jointly stratified target population with geographic region, time horizon, and funding source; vaccine-type strata were defined within population. | To reduce confounding by target population and ensure that subgroup comparisons were clinically interpretable. |

1. Wan X, Wang W, Liu J, Tong T. Estimating the sample mean and standard deviation from the sample size, median, range and/or interquartile range. BMC Med Res Methodol. 2014;14:135. https://doi.org/10.1186/1471-2288-14-135.
