# Supplementary material for: Cost-effectiveness of respiratory syncytial virus vaccines in post-marketing settings: a systematic review and meta-analysis
Source: Infect Dis Poverty. 2026 Nov 14;15:117. doi: 10.1186/s40249-026-01520-y (PMC13770325; doi:10.1186/s40249-026-01520-y)
Supplement: Supplementary file 3 [file 40249_2026_1520_MOESM3_ESM.docx]

# Supplementary material 3. Search strategy

**PubMed**

| Search number | Query |
| --- | --- |
| #1  RSV-related | (RSV[Title/Abstract]) OR ((("Respiratory Syncytial Virus Infections"[Mesh] OR Respiratory Syncytial Virus Infection OR Infections, Respiratory Syncytial Virus OR RSV Infection OR Infection, RSV OR RSV Infections) OR ("Respiratory Syncytial Virus, Human"[Mesh] OR Human respiratory syncytial virus OR HRSV Human respiratory syncytial virus OR human RSV OR human RSVs OR RSV, human)) OR ("Respiratory Syncytial Viruses"[Mesh] OR Respiratory Syncytial Virus OR Syncytial Virus, Respiratory OR Virus, Respiratory Syncytial OR Orthopneumovirus OR Orthopneumoviruses OR RSV Respiratory Syncytial Virus OR Chimpanzee Coryza Agent OR Chimpanzee Coryza Agents OR Coryza Agent, Chimpanzee)) |
| #2  cost-effectiveness-related | (cost*[Title/Abstract]) OR ((("Cost-Effectiveness Analysis"[Mesh] OR Analysis, Cost-Effectiveness OR Cost Effectiveness Analysis OR Cost Effectiveness OR Effectiveness, Cost OR Cost Effectiveness Ratio OR Cost Effectiveness Ratios OR Effectiveness Ratio, Cost OR Ratio, Cost Effectiveness) OR ("Cost-Benefit Analysis"[Mesh] OR Analysis, Cost-Benefit OR Cost-Benefit Analyses OR Cost Benefit Analysis OR Analyses, Cost Benefit OR Analysis, Cost Benefit OR Cost Benefit Analyses OR Cost Benefit OR Cost and Benefit OR Benefit and Cost OR Benefits and Costs OR Costs and Benefits OR Cost-Benefit Data OR Cost Benefit Data OR Data, Cost-Benefit OR Cost-Utility Analysis OR Analysis, Cost-Utility OR Cost-Utility Analyses OR Cost Utility Analysis OR Marginal Analysis OR Analysis, Marginal OR Marginal Analyses OR Economic Evaluation OR Economic Evaluations OR Evaluation, Economic)) OR ("Economics, Pharmaceutical"[Mesh] OR Pharmacoeconomics OR Pharmaceutical Economics OR Pharmacy Economics OR Economic, Pharmacy OR Economics, Pharmacy OR Pharmacy Economic)) |
| #3  Vaccine-related | (vaccin*[Title/Abstract]) OR ("Respiratory Syncytial Virus Vaccines"[Mesh] OR RSV Vaccine OR Vaccine, RSV OR Respiratory Syncytial Virus Vaccine OR RSV Vaccines OR Vaccines, RSV) |
| #4 | #1 AND #2 AND #3 |

**Embase**

| Search number | Query |
| --- | --- |
| #1  RSV-related | 'respiratory syncytial virus infection'/exp  OR 'respiratory syncytial virus'/exp  OR rsv:ti,ab,kw  OR 'respiratory syncytial virus':ti,ab,kw  OR 'respiratory syncytial virus infection':ti,ab,kw  OR 'human respiratory syncytial virus':ti,ab,kw  OR hrsv:ti,ab,kw  OR orthopneumovirus*:ti,ab,kw  OR 'chimpanzee coryza agent':ti,ab,kw |
| #2  cost-effectiveness-related | 'cost effectiveness analysis'/exp  OR 'cost benefit analysis'/exp  OR 'cost utility analysis'/exp  OR 'economic evaluation'/exp  OR 'pharmacoeconomics'/exp  OR cost*:ti,ab,kw  OR 'cost effectiveness':ti,ab,kw  OR 'cost effective':ti,ab,kw  OR 'cost utility':ti,ab,kw  OR 'cost benefit':ti,ab,kw  OR 'economic evaluation':ti,ab,kw  OR 'economic evaluations':ti,ab,kw  OR pharmacoeconomic*:ti,ab,kw  OR icer:ti,ab,kw  OR 'incremental cost effectiveness ratio':ti,ab,kw |
| #3  Vaccine-related | 'respiratory syncytial virus vaccine'/exp  OR 'vaccine'/exp  OR vaccin*:ti,ab,kw  OR 'rsv vaccine':ti,ab,kw  OR 'rsv vaccines':ti,ab,kw  OR 'respiratory syncytial virus vaccine':ti,ab,kw  OR 'respiratory syncytial virus vaccines':ti,ab,kw |
| #4：Final combined search | #1 AND #2 AND #3 |

**Cochrane**

| Search number | Query |
| --- | --- |
| #1 | MeSH descriptor: [Respiratory Syncytial Virus Infections] explode all trees |
| #2 | MeSH descriptor: [Respiratory Syncytial Viruses] explode all trees |
| #3 | MeSH descriptor: [Respiratory Syncytial Virus, Human] explode all trees |
| #4 | (RSV OR "respiratory syncytial virus" OR "respiratory syncytial virus infection" OR "human respiratory syncytial virus" OR HRSV OR "human RSV" OR "human RSVs"):ti,ab,kw |
| #5 | #1 OR #2 OR #3 OR #4 |
| #6 | MeSH descriptor: [Cost-Benefit Analysis] explode all trees |
| #7 | MeSH descriptor: [Costs and Cost Analysis] explode all trees |
| #8 | MeSH descriptor: [Economics, Pharmaceutical] explode all trees |
| #9 | (cost* OR "cost effectiveness" OR "cost-effectiveness" OR "cost effective" OR "cost-effective" OR "cost utility" OR "cost-utility" OR "cost benefit" OR "cost-benefit" OR "economic evaluation" OR "economic evaluations" OR pharmacoeconomic* OR ICER):ti,ab,kw |
| #10 | #6 OR #7 OR #8 OR #9 |
| #11 | MeSH descriptor: [Respiratory Syncytial Virus Vaccines] explode all trees |
| #12 | MeSH descriptor: [Vaccines] explode all trees |
| #13 | (vaccin* OR immunization OR immunisation OR immuniz* OR immunis* OR "RSV vaccine" OR "RSV vaccines" OR "respiratory syncytial virus vaccine" OR "respiratory syncytial virus vaccines"):ti,ab,kw |
| #14 | #11 OR #12 OR #13 |
| #15 | #5 AND #10 AND #14 |

**Web of Science**

| Search number | Query |
| --- | --- |
| #1 | TS=(RSV OR "respiratory syncytial virus" OR "respiratory syncytial virus infection*" OR "human respiratory syncytial virus" OR HRSV OR "human RSV" OR "human RSVs") |
| #2 | TS=(cost* OR "cost effectiveness" OR "cost-effectiveness" OR "cost effective" OR "cost-effective" OR "cost effectiveness analysis" OR "cost-effectiveness analysis" OR "cost effectiveness ratio*" OR "cost-effectiveness ratio*" OR "cost benefit" OR "cost-benefit" OR "cost benefit analysis" OR "cost-benefit analysis" OR "cost utility" OR "cost-utility" OR "cost utility analysis" OR "cost-utility analysis" OR "economic evaluation" OR "economic evaluations" OR pharmacoeconomic* OR "pharmaceutical economic*" OR "pharmacy economic*" OR ICER OR "incremental cost effectiveness ratio" OR "incremental cost-effectiveness ratio") |
| #3 | TS=(vaccin* OR immunization OR immunisation OR immuniz* OR immunis* OR "RSV vaccine" OR "RSV vaccines" OR "respiratory syncytial virus vaccine" OR "respiratory syncytial virus vaccines") |
| #4 | #1 AND #2 AND #3 |

**Standard grey literature search terms**

Concept #1: RSV-related terms

("respiratory syncytial virus" OR RSV OR "RSV infection" OR "RSV disease" OR "human respiratory syncytial virus" OR HRSV OR orthopneumovirus)

AND

Concept #2: Vaccine-related terms

(vaccine OR vaccination OR immunization OR immunisation OR RSVpreF OR RSVPreF3 OR "prefusion F vaccine" OR "bivalent RSV prefusion F" OR Abrysvo OR Arexvy)

AND

Concept #3: Economic evaluation-related terms

("cost-effectiveness" OR "cost effectiveness" OR "cost-utility" OR "cost utility" OR "cost-benefit" OR "cost benefit" OR "economic evaluation" OR pharmacoeconomic OR pharmacoeconomics OR ICER)

Search results with the terms above

**INAHTA International Health Technology Assessment (HTA) Database**: *n* = 1

**Google Scholar**: *n* = 100 (screened among the top 100 most relevant results).

Highly simplified grey literature search terms

("RSV" OR "respiratory syncytial virus") AND cost* AND vaccin*

Search results with the terms above

**CDA-AMC reports**: *n* = 1 (Select Report Type: View All Reports)

**WHO IRIS**: *n* = 188 (limited to publications from 2020 to 2025)

C**DC ACIP meeting slides**: *n* = 13 under the “RSV vaccine” topic filter
