# Supplementary material for: Cost-effectiveness of respiratory syncytial virus vaccines in post-marketing settings: a systematic review and meta-analysis
Source: Infect Dis Poverty. 2026 Nov 14;15:117. doi: 10.1186/s40249-026-01520-y (PMC13770325; doi:10.1186/s40249-026-01520-y)
Supplement: Supplementary file 4 [file 40249_2026_1520_MOESM4_ESM.docx]

# Supplementary material 4. Scenario for data preparation

Meta-analysis (random-effects model)

$$INBp=\frac{\sum_{i=1}^{s} w_{i}.INBi}{\sum_{i=1}^{s} w_{i}}$$

$$w_{i}=\frac{1}{var \left( INBi \right)+ \tau^{2}}$$

$$\tau^{2}=\frac{Q-(S-1)}{\sum w_{i}- \frac{\sum w_{i}^{2}}{\sum w_{i}} \tau^{2}}$$

Q is the Cochrane Q-statistic, where Q = 0 if Q < S-1; and s is the number of included studies/ comparison

The mean of INB was calculated using the following equations:

$INB=\Delta E\left( K-ICER \right)$

$INB=\left( K\times\Delta E \right)-\Delta C$

The variance of INB was estimated using the following equation.

$\sigma_{INB}^{2}$ = $K^{2}\times\sigma_{\Delta E}^{2}+\sigma_{\Delta C}^{2}-2\times K\times\rho_{\Delta E\Delta C}\times\sigma_{\Delta E}\times\sigma_{\Delta C}$

A common workaround is to digitize published PSA scatter plots using image-based extraction tools. However, due to severe point overlap in dense regions of the plot, digitization procedures tend to recover only a subset of visually distinguishable points, resulting in a biased and incomplete representation of the original PSA sample.

Under the assumption that the original PSA scatter points and the digitized points obtained from image-based extraction tools follow elliptically contoured distributions with the same location parameter and shape (scatter) matrix, but different radial density functions, both datasets share the same correlation structure between incremental costs ($\Delta C$) and incremental effects ($\Delta E$). Therefore, the correlation estimated from the digitized points is used as a proxy for the correlation of the original PSA scatter.

**(a) Scenarios for data preparation**

Scenario 1

When studies reported 95% *CIs* for ΔC and ΔE, the SDs of ΔC and ΔE were derived using Equation (1). The variance of INB was then estimated according to Equation (2).

$\sigma=\frac{upper limit of 95\% CI-lower limit of 95\% CI}{2\times1.96}\times\sqrt{N}$	(1)

$\sigma_{INB}^{2}$ = $K^{2}\times\sigma_{\Delta E}^{2}+\sigma_{\Delta C}^{2}-2\times K\times\rho_{\Delta E\Delta C}\times\sigma_{\Delta E}\times\sigma_{\Delta C}$	(2)

where N represents the sample size and $\rho_{\Delta E\Delta C}\mathrm{represents}$ the correlation between $\Delta E$ and $\Delta C$ derived from digitized points using WebPlotDigitizer (version 4.4).

Scenario 2

When studies did not report variances or 95% *CIs* but presented scatter plots of ΔC and ΔE from PSA, the variance of INB was estimated based on these plots. Because substantial overlap among scatter points made direct extraction of all data infeasible, we instead used WebPlotDigitizer (version 4.4) to extract the maximum and minimum values of ΔE and ΔC from the PSA scatter plots. The SD was then estimated following the method proposed by Wan et al.

Specifically, under the assumption that the underlying data are approximately normally distributed, the SD can be consistently approximated using order-statistic properties of the normal distribution. Let $a$ and $b$ denote the minimum and maximum values and $n$ the sample size.

When only the range is available $\left\{ a , b , n \right\}$, the SD is estimated as

$$\hat{\sigma}\approx\frac{b-a}{2\Phi^{-1}\text{ }\left( \frac{n-0.375}{\text{ }n+0.25\text{ }} \right)},$$

where $\Phi^{-1}(\cdot)$ denotes the inverse standard normal cumulative distribution function. The variance of INB was then estimated according to Equation (2).

**(b) Currency conversion**

All monetary outcomes were converted to 2023 USD using Turner et al.’s adjustment method 1.

The conversion factor was calculated as:

$$R=\frac{1}{ER_{t}}\times\frac{GDPD_{US,2023}}{GDPD_{US,t}}$$

Costing data, including $\Delta C$, ICER, WTP threshold $\left( K \right)$, and INB, were converted using:

$$Y_{USD,2023}=Y_{local,t}\times R$$

SDs of monetary outcomes were converted using:

$$SD_{USD,2023}=SD_{local,t}\times R$$

where $Y_{local,t}$ is the monetary value reported in the study currency in costing year $t$; $ER_{t}$ is the exchange rate in costing year $t$, expressed as local currency units per USD; $GDPD_{US,t}$ and $GDPD_{US,2023}$ are the GDP implicit price deflators for the United States in year $t$ and 2023, respectively; and $R$ is the conversion factor.
