# Supplementary material for: Cost-effectiveness of respiratory syncytial virus vaccines in post-marketing settings: a systematic review and meta-analysis
Source: Infect Dis Poverty. 2026 Nov 14;15:117. doi: 10.1186/s40249-026-01520-y (PMC13770325; doi:10.1186/s40249-026-01520-y)
Supplement: Supplementary file 5 [file 40249_2026_1520_MOESM5_ESM.docx]

# Supplementary material 5. Studies excluded after full-text assessment

**Table S1. Selected potentially eligible studies and related reports excluded after full-text assessment.**

| **Study** | **Principal reason for exclusion** |
| --- | --- |
| **Gessner (2000)**  The cost-effectiveness of a hypothetical respiratory syncytial virus vaccine in the elderly  https://doi.org/10.1016/s0264-410x(99)00425-9 | Evaluated a hypothetical RSV vaccine with an assumed price, rather than a marketed vaccine with an established local price. |
| **Meijboom et al. (2012)**  Cost-effectiveness of potential infant vaccination against respiratory syncytial virus infection in The Netherlands  https://doi.org/10.1016/j.vaccine.2012.04.072 | Evaluated a candidate infant RSV vaccine using assumed effectiveness and a fixed assumed price of EUR 37.50 per dose. |
| **Meijboom et al. (2013)**  RSV vaccine in development: Assessing the potential cost-effectiveness in the Dutch elderly population  https://doi.org/10.1016/j.vaccine.2013.10.023 | Evaluated a hypothetical vaccine and maximum affordable vaccination costs, rather than a marketed vaccine at an established local price. |
| **Pouwels et al. (2016)**  Potential cost-effectiveness of RSV vaccination of infants and pregnant women in Turkey: an illustration based on Bursa data  https://doi.org/10.1371/journal.pone.0163567 | Evaluated hypothetical infant and maternal RSV vaccination, with an assumed vaccine price of TL 60 per dose. |
| **Cromer et al. (2017)**  Burden of paediatric respiratory syncytial virus disease and potential effect of different immunisation strategies: a modelling and cost-effectiveness analysis for England  https://doi.org/10.1016/S2468-2667(17)30103-2 | Estimated maximum cost-effective prices for prospective immunisation strategies, rather than cost-effectiveness at established local vaccine prices. |
| **Hodgson et al. (2020)**  Evaluating the next generation of RSV intervention strategies: a mathematical modelling study and cost-effectiveness analysis  https://doi.org/10.1186/s12916-020-01802-8 | Estimated maximum purchasing prices for next-generation RSV interventions rather than evaluating marketed vaccination at established local prices. |
| **Li et al. (2020)**  Health and economic burden of respiratory syncytial virus (RSV) disease and the cost-effectiveness of potential interventions against RSV among children under 5 years in 72 Gavi-eligible countries  https://doi.org/10.1186/s12916-020-01537-6 | Evaluated prospective maternal RSV vaccination using assumed costs, without an established local RSV vaccine price. |
| **Baral et al. (2021)**  Impact and cost-effectiveness of potential interventions against infant respiratory syncytial virus (RSV) in 131 low-income and middle-income countries using a static cohort model  https://doi.org/10.1136/bmjopen-2020-046563 | Evaluated candidate maternal RSV vaccination using assumed prices of USD 3 or USD 5 per dose, rather than established local vaccine prices. |
| **Laufer et al. (2021)**  Cost-effectiveness of infant respiratory syncytial virus preventive interventions in Mali: a modeling study to inform policy and investment decisions  https://doi.org/10.1016/j.vaccine.2021.06.086 | Evaluated candidate RSV prevention products in a pre-marketing setting using assumed product prices. |
| **Liu et al. (2021)**  Cost-effectiveness of strategies for preventing paediatric lower respiratory infections associated with respiratory syncytial virus in eight Chinese cities  https://doi.org/10.1016/j.vaccine.2021.08.057 | Reported threshold strategy costs for prospective interventions, rather than cost-effectiveness at established local vaccine prices. |
| **Nourbakhsh et al. (2021)**  Effectiveness and cost-effectiveness of RSV infant and maternal immunization programs: a case study of Nunavik, Canada  https://doi.org/10.1016/j.eclinm.2021.101141 | Evaluated the investigational maternal vaccine ResVax rather than a marketed RSV vaccine. |
| **Herring et al. (2022)**  Clinical and economic outcomes associated with respiratory syncytial virus vaccination in older adults in the United States  https://doi.org/10.1016/j.vaccine.2021.12.002 | Evaluated a hypothetical RSV vaccine and its value-based price, rather than a marketed vaccine at an established local price. |
| **Li et al. (2022)**  Cost-effectiveness of respiratory syncytial virus disease prevention strategies: maternal vaccine versus seasonal or year-round monoclonal antibody program in Norwegian children  https://doi.org/10.1093/infdis/jiac064 | Used an assumed maternal vaccine price of NOK 500 per dose, based on a rotavirus vaccine evaluation because manufacturer pricing was unavailable. |
| **Zeevat et al. (2022)**  Exploratory analysis of the economically justifiable price of a hypothetical RSV vaccine for older adults in the Netherlands and the United Kingdom  https://doi.org/10.1093/infdis/jiab118 | Estimated economically justifiable prices for a hypothetical RSV vaccine, rather than evaluating an established local market price. |
| **Li et al. (2023)**  Cost-effectiveness of respiratory syncytial virus preventive interventions in children: a model comparison study  https://doi.org/10.1016/j.jval.2022.11.014 | Compared models in a hypothetical country setting using a common assumed input set, rather than evaluating a specific post-marketing setting. |
| **Koltai et al. (2023)**  Estimating the cost-effectiveness of maternal vaccination and monoclonal antibodies for respiratory syncytial virus in Kenya and South Africa  https://doi.org/10.1186/s12916-023-02806-w | Evaluated maternal vaccination over assumed price scenarios of USD 5-50 per course, rather than using established local vaccine prices. |
| **Mahmud et al. (2023)**  Cost-effectiveness of pharmaceutical strategies to prevent respiratory syncytial virus disease in young children: a decision-support model for use in low-income and middle-income countries  https://doi.org/10.1186/s12916-023-02827-5 | Used assumed maternal vaccine prices of USD 3.50 or USD 7 per dose in illustrative country applications, rather than established local vaccine prices. |
| **Laufer et al. (2023)**  Optimizing next-generation RSV prevention in Mali: a cost-effectiveness analysis of pediatric vaccination, maternal vaccination, and extended half-life monoclonal antibody immunoprophylaxis  https://doi.org/10.1371/journal.pgph.0001432 | Evaluated candidate interventions using an assumed base-case price of USD 1 per dose, derived from the price of a pentavalent vaccine. |
| **Srikantiah and Klugman (2023)**  New respiratory syncytial virus immunization products in low- and middle-income countries: potential for cost-effective impact on a high burden of disease in young infants  https://doi.org/10.1186/s12916-023-02883-x | Editorial discussing other studies; not an original full economic evaluation. |
| **Postma et al. (2023)**  Predicted public health and economic impact of respiratory syncytial virus vaccination with variable duration of protection for adults ≥ 60 years in Belgium  https://doi.org/10.3390/vaccines11050990 | Evaluated a generic candidate vaccine and treatment-cost savings; did not provide a full cost-effectiveness evaluation of a marketed vaccine. |
| **Wang et al. (2023)**  Comparative cost-effectiveness analysis of respiratory syncytial virus vaccines for older adults in Hong Kong  https://doi.org/10.3390/vaccines11101605 | RSV vaccines were not yet marketed in Hong Kong at evaluation; prices were benchmarked at 25-100% of United States vaccine prices. |
| **Do et al. (2023)**  Impact and cost-effectiveness of strategies to prevent respiratory syncytial virus (RSV) disease in Vietnam: a modelling study  https://doi.org/10.1016/j.vaccine.2023.09.003 | Evaluated prospective maternal vaccination using assumed dose prices, including USD 25 in the central scenario, rather than an established local vaccine price. |
| **Shoukat et al. (2023)**  Cost-effectiveness analysis of nirsevimab and maternal RSVpreF vaccine strategies for prevention of Respiratory Syncytial Virus disease among infants in Canada: a simulation study  https://doi.org/10.1016/j.lana.2023.100629 | Determined maximum cost-effective prices per dose rather than evaluating maternal vaccination at an established local vaccine price. |
| **Hodgson et al. (2024)**  Protecting infants against RSV disease: an impact and cost-effectiveness comparison of long-acting monoclonal antibodies and maternal vaccination  https://doi.org/10.1016/j.lanepe.2023.100829 | Compared programmes across assumed procurement and administration cost scenarios rather than established local vaccine prices. |
| **Shoukat et al. (2024)**  Impact and cost-effectiveness analyses of vaccination for prevention of respiratory syncytial virus disease among older adults in Ontario: A Canadian Immunization Research Network (CIRN) study  https://doi.org/10.1016/j.vaccine.2024.02.041 | Determined maximum cost-effective prices per dose for older-adult vaccination rather than using established local vaccine prices. |
| **Moghadas et al. (2024)**  Cost-effectiveness of prefusion F protein-based vaccines against respiratory syncytial virus disease for older adults in the United States  https://doi.org/10.1093/cid/ciad658 | Estimated maximum cost-effective prices per dose for Arexvy and Abrysvo rather than evaluating established local vaccine prices. |
| **Neri et al. (2024)**  Impact of including productivity costs in economic analyses of vaccines for C. difficile infections and infant respiratory syncytial virus, in a UK setting  https://doi.org/10.1186/s12962-024-00533-4 | Assessed productivity-cost savings without including vaccine costs or incremental health gains; insufficient information for the required INB outcome. |
| **Ishiwada et al. (2024)**  Cost-effectiveness analysis of maternal respiratory syncytial virus vaccine in protecting infants from RSV infection in Japan  https://doi.org/10.1007/s40121-024-01000-6 | Explored assumed vaccine prices and maximum cost-effective prices, rather than using an established local RSVpreF price. |
| **Guiñazú et al. (2024)**  Evaluation of the potential impact and cost-effectiveness of respiratory syncytial virus (RSV) prevention strategies for infants in Argentina  https://doi.org/10.1016/j.vaccine.2024.126234 | Evaluated assumed vaccine price scenarios of USD 25, USD 50, and USD 100 per dose rather than an established local vaccine price. |
| **Mizukami et al. (2024)**  Cost-effectiveness analysis of respiratory syncytial virus vaccination with the adjuvanted prefusion F protein vaccine (RSVPreF3 OA) for adults ≥ 60 years old in Japan  https://doi.org/10.1080/14760584.2024.2410898 | Assumed a vaccine price of JPY 20,000 per dose, rather than using an established local price. |
| **Rey-Ares et al. (2024)**  Cost-effectiveness of bivalent respiratory syncytial virus prefusion F (RSVpreF) vaccine during pregnancy for prevention of respiratory syncytial virus among infants in Argentina  https://doi.org/10.1007/s40121-024-01055-5 | Estimated the value-based price of maternal RSVpreF vaccination rather than evaluating cost-effectiveness at an established local vaccine price. |
| **Huerta et al. (2025)**  Economic and clinical benefits of bivalent respiratory syncytial virus prefusion F (RSVpreF) maternal vaccine for prevention of RSV in infants: a cost-effectiveness analysis for Mexico  https://doi.org/10.3390/vaccines13010077 | The local vaccine unit cost was undetermined; the analysis estimated cost-saving and cost-effective prices per dose. |
| **Nazareno et al. (2025)**  Estimating the cost-effectiveness of maternal respiratory syncytial virus (RSV) vaccination in Australia: a dynamic and economic modelling analysis  https://doi.org/10.1016/j.vaccine.2024.126651 | Assumed a base-case vaccine price of AUD 75 per dose because the Australian price was unknown, and explored threshold prices. |
| **Bugden et al. (2025)**  Cost-effectiveness of nirsevimab and maternal RSVpreF for preventing respiratory syncytial virus disease in infants across Canada  https://doi.org/10.1186/s12916-025-03928-z | Used United States CDC contract prices converted to Canadian dollars because Canadian drug costing information was unavailable; no established local vaccine price. |
| **Li et al. (2025)**  Influential drivers of the cost-effectiveness of respiratory syncytial virus vaccination in European older adults: a multi-country analysis  https://doi.org/10.1186/s12916-025-03970-x | Evaluated a hypothetical vaccine profile with an assumed common price of EUR 150 per dose across countries, rather than established local vaccine prices. |
| **Klimek (2025)**  Cost effectiveness of vaccinations: on the complexity of health economic analyses of influenza, SARS-CoV-2 and RSV vaccination  https://doi.org/10.1007/s00103-025-04022-8 | German-language narrative review rather than an English-language original economic evaluation. |
