# Supplementary material for: Cost-effectiveness of respiratory syncytial virus vaccines in post-marketing settings: a systematic review and meta-analysis
Source: Infect Dis Poverty. 2026 Nov 14;15:117. doi: 10.1186/s40249-026-01520-y (PMC13770325; doi:10.1186/s40249-026-01520-y)
Supplement: Supplementary file 6 [file 40249_2026_1520_MOESM6_ESM.docx]

# Supplementary material 6**. Assessment of risk of** bias

| **Author (publication year)** | **La (2024)** | **Averin (2025; Germany)** | **Hutton (2024)** | **Szilcz (2025)** | **Waize (2025)** | **Gourzoulidis (2024)** | **Álvarez Aldean (2024)** | **Averin (2025; United States)** | **Wang (2025)** | **Tuite (2024)** | **Gourzoulidis (2025)** | **Nguyen (2025)** | **Rudd (2025)** | **Li (2025)** | **Værnø (2025)** | **Singer (2025)** | **Hutton (2024; maternal)** |
| --- | --- | --- | --- | --- | --- | --- | --- | --- | --- | --- | --- | --- | --- | --- | --- | --- | --- |
| Narrow perspective bias | Yes | Yes | Yes | Yes | Partly | Partly | Partly | Yes | Yes | Partly | Partly | Yes | Partly | Yes | Yes | Yes | Yes |
| Inefficient comparator bias | Yes | Yes | Yes | Yes | Yes | Yes | Yes | Yes | Yes | Yes | Yes | Yes | Yes | Yes | Partly | Yes | Yes |
| Cost measurement/omission bias | Yes | Yes | Yes | Yes | Yes | Yes | Yes | Yes | Yes | Yes | Yes | Yes | Yes | Yes | Yes | Yes | Yes |
| Intermittent data collection bias | Yes | Yes | Yes | Yes | Yes | Yes | Yes | Unclear | Yes | Yes | Yes | Yes | Yes | Yes | Yes | Yes | Yes |
| Invalid valuation bias | Yes | Yes | Yes | Yes | Yes | Yes | Yes | Yes | Yes | Yes | Yes | Yes | Yes | Partly | Yes | Yes | Yes |
| Ordinal ICER bias | Yes | Yes | Yes | Yes | Yes | Yes | Yes | Yes | Yes | Yes | Yes | Yes | Yes | Yes | Yes | Yes | Yes |
| Double counting bias | Yes | Yes | Yes | Yes | Yes | Yes | Yes | Yes | Yes | Yes | Yes | Yes | Yes | Yes | Yes | Yes | Yes |
| Inappropriate discounting bias | Yes | Yes | Yes | Yes | Yes | Partly | Yes | Yes | Yes | Partly | Yes | Yes | Partly | Yes | Yes | Yes | Yes |
| Limited sensitivity analysis bias | Yes | Yes | Yes | Yes | Yes | Yes | Yes | Yes | Yes | Partly | Partly | Partly | No | Yes | Yes | Yes | Yes |
| Sponsor bias | Partly | Partly | Yes | Partly | Partly | Partly | Partly | Partly | Yes | Yes | Partly | Yes | Yes | Yes | Partly | Partly | Yes |
| Reporting & dissemination bias | Unclear | Unclear | Unclear | Unclear | Unclear | Unclear | Unclear | Unclear | Unclear | Unclear | Unclear | Unclear | Unclear | Unclear | Unclear | Unclear | Unclear |
| Structural assumption bias | Yes | Partly | Partly | Yes | Yes | Partly | Yes | Partly | Partly | Yes | Partly | Partly | Yes | Yes | Partly | Yes | Partly |
| No-treatment comparator bias | Yes | Yes | Yes | Yes | Yes | Yes | Yes | Yes | Yes | Yes | Yes | Yes | Yes | Yes | Yes | Yes | Yes |
| Wrong model bias | Yes | Yes | Yes | Yes | Yes | Yes | Yes | Yes | Yes | Yes | Yes | Yes | Yes | Yes | Yes | Yes | Yes |
| Limited time horizon bias | Yes | Yes | Partly | Yes | Yes | Yes | Partly | Yes | Partly | Partly | Yes | Yes | Partly | Yes | Partly | Yes | Partly |
| Bias related to data identification | Yes | Yes | Yes | Partly | Yes | Yes | Yes | Partly | Yes | Yes | Partly | Yes | Yes | Yes | Yes | Yes | Yes |
| Bias related to baseline data | Yes | Yes | Yes | Partly | Yes | Partly | Yes | Yes | Yes | Yes | Yes | Partly | Yes | Yes | Partly | Yes | Yes |
| Bias related to treatment effects | Yes | Yes | Partly | Yes | Yes | Yes | Yes | Yes | Yes | Yes | Yes | Yes | Yes | Yes | Yes | Yes | Yes |
| Bias related to quality-of-life weights (utilities) | Yes | Yes | Yes | Yes | Yes | Yes | Yes | Yes | Yes | Yes | Yes | Yes | Yes | Yes | Yes | Yes | Yes |
| Non-transparent data incorporation bias | Yes | Yes | Yes | Yes | Yes | Yes | Yes | Partly | Yes | Yes | Yes | Yes | Partly | Yes | Yes | Yes | Yes |
| Limited scope bias | Yes | Yes | Yes | Yes | Yes | Yes | Yes | Yes | Yes | Partly | Partly | Yes | Partly | Yes | Yes | Yes | Yes |
| Bias related to internal consistency | Unclear | Unclear | Unclear | Unclear | Unclear | Unclear | Unclear | Unclear | Unclear | Unclear | Unclear | Unclear | Unclear | Partly | Unclear | Unclear | Unclear |
| **Overall risk of bias** | **Low** | **Low** | **Moderate** | **Low** | **Low** | **Low** | **Low** | **Low** | **Low** | **Moderate** | **Moderate** | **Moderate** | **High** | **Low** | **Low** | **Low** | **Low** |

Note: Yes indicates that the ECOBIAS criterion was adequately addressed; Partly, Unclear, and No indicate increasing concern or insufficient reporting. Overall risk was classified from limited sensitivity analysis bias, wrong model bias, and bias related to treatment effects: all Yes=low risk; any Partly/Unclear=moderate risk; any No=high risk.
