# Supplementary material for: Cost-effectiveness of respiratory syncytial virus vaccines in post-marketing settings: a systematic review and meta-analysis
Source: Infect Dis Poverty. 2026 Nov 14;15:117. doi: 10.1186/s40249-026-01520-y (PMC13770325; doi:10.1186/s40249-026-01520-y)
Supplement: Supplementary file 7 [file 40249_2026_1520_MOESM7_ESM.docx]

# Supplementary material 7. Assessment of publication bias

Publication-bias risk was explored separately within each population using funnel plots and Egger's regression test. Because Hutton reported two correlated vaccine scenarios, older-adult analyses retained one Hutton scenario at a time. Each analysis included only 5-6 studies, below the commonly recommended minimum of 10; funnel-plot asymmetry and Egger tests should therefore be interpreted cautiously as exploratory findings.

*Figure S1. Funnel plots of pooled INB by population and Hutton vaccine scenario.*

**Table S2. Egger regression tests for funnel-plot asymmetry.**

| **Population/scenario** | ***k*** | **Egger t statistic** | ***P* value** |
| --- | --- | --- | --- |
| Infants | 5 | 0.06 | 0.953 |
| Older adults: Hutton-Abrysvo retained | 6 | 4.19 | 0.014 |
| Older adults: Hutton-Arexvy retained | 6 | 4.12 | 0.015 |

Note: Egger's test did not indicate asymmetry for infants (*P* = 0.953), whereas both older-adult scenarios indicated asymmetry (*P* = 0.014 and *P* = 0.015). These results should not be treated as definitive evidence of publication bias because each analysis contained fewer than 10 studies and the tests have low power and unstable type I error in small samples. INB, incremental net benefit; k, number of studies.

**Contour-enhanced funnel plot assessment**

Because Egger's tests indicated funnel-plot asymmetry in both older-adult scenarios, contour-enhanced funnel plots were constructed around the null effect (INB = 0), with two-sided significance contours at *P* = 0.10, *P* = 0.05, and *P* = 0.01. A pattern in which the sparsely populated side lies mainly in non-significant regions is more compatible with publication bias; gaps extending across significant regions suggest that between-study heterogeneity or other small-study effects may also explain the asymmetry.

*Figure S2. Contour-enhanced funnel plots for the two older-adult Hutton scenarios.*

Only six studies were available in each older-adult scenario; the contour-based assessment is therefore exploratory and cannot determine the cause of asymmetry with certainty.
