# Supplementary material for: Cost-effectiveness of respiratory syncytial virus vaccines in post-marketing settings: a systematic review and meta-analysis
Source: Infect Dis Poverty. 2026 Nov 14;15:117. doi: 10.1186/s40249-026-01520-y (PMC13770325; doi:10.1186/s40249-026-01520-y)
Supplement: Supplementary file 8 [file 40249_2026_1520_MOESM8_ESM.docx]

# Supplementary material 8. Sensitivity analyses

Leave-one-out sensitivity analyses were performed separately within each population using DerSimonian-Laird random-effects models. Because Hutton reported two correlated vaccine scenarios, the older-adult analysis was run twice, retaining one Hutton scenario at a time. The infant pooled estimate was sensitive to exclusion of Wang, whereas the pooled INB remained positive after every single-study exclusion in both older-adult scenarios.

**Table S3. Leave-one-study-out sensitivity analysis for infants (5 studies)**

| **Study excluded** | **Pooled INB (95% *CI*)** | ***I*² (%)** |
| --- | --- | --- |
| Averin et al. (2025), United States | -6.81 [-51.01; 37.39] | 90.5 |
| Álvarez Aldean et al. (2024), Spain | -3.87 [-51.03; 43.30] | 90.8 |
| Værnø et al. (2025), Norway | 7.09 [-52.50; 66.68] | 92.1 |
| Li et al. (2025), Belgium | 5.31 [-56.71; 67.33] | 91.8 |
| Wang et al. (2025), Hong Kong, China | 23.98 [0.45; 47.51] | 60.5 |

**Table S4. Leave-one-study-out analysis for older adults (Hutton-Abrysvo retained; 6 studies)**

| **Study excluded** | **Pooled INB (95% *CI*)** | ***I*² (%)** |
| --- | --- | --- |
| La et al. (2024), United States | 84.81 [23.56; 146.07] | 93.6 |
| Hutton et al. (2024), United States | 151.33 [65.90; 236.76] | 88.9 |
| Szilcz et al. (2025), Sweden | 55.82 [3.45; 108.19] | 89.8 |
| Waize et al. (2025), Germany | 96.62 [30.83; 162.42] | 94.1 |
| Gourzoulidis et al. (2024), Greece | 135.62 [45.95; 225.28] | 92.8 |
| Averin et al. (2025), Germany | 138.36 [55.09; 221.63] | 94.4 |

**Table S5. Leave-one-study-out analysis for older adults (Hutton-Arexvy retained; 6 studies)**

| **Study excluded** | **Pooled INB (95% *CI*)** | ***I*² (%)** |
| --- | --- | --- |
| La et al. (2024), United States | 85.13 [22.15; 148.11] | 93.9 |
| Hutton et al. (2024), United States | 151.33 [65.90; 236.76] | 88.9 |
| Szilcz et al. (2025), Sweden | 56.45 [2.05; 110.86] | 90.5 |
| Waize et al. (2025), Germany | 97.32 [29.78; 164.87] | 94.4 |
| Gourzoulidis et al. (2024), Greece | 135.75 [44.55; 226.94] | 93.0 |
| Averin et al. (2025), Germany | 138.87 [53.74; 224.01] | 94.6 |

*Note: Each row reports the random-effects pooled INB after excluding the specified study. I² denotes residual heterogeneity. In Tables S4 and S5, excluding Hutton removes the retained Hutton scenario.*

**Sensitivity analysis including Singer et al.**

Singer et al. evaluated a distinct, narrowly risk-enriched population of adults in the United States aged 50–59 years with chronic obstructive pulmonary disease and increased risk of severe RSV disease. The study was excluded from the primary pooled estimate because its target population differed from the predominantly age-eligible adult populations, and was added here to assess the influence of that decision. Singer et al. reported an INB of 850.56 (95% *CI*: 73.07 to 1628.06) and received approximately 0.7% weight. With Hutton-Abrysvo retained, the pooled adult INB was 111.34 (95% *CI*: 47.10 to 175.57; *I*² = 92.3%); with Hutton-Arexvy retained, it was 112.26 (95% *CI*: 46.49 to 178.03; *I*² = 92.6%). The direction and statistical significance of the pooled adult estimates were unchanged.

*Figure S3. Population-specific random-effects meta-analysis of INB after including Singer et al. Panel A shows the older-adult analysis retaining the Hutton-Abrysvo estimate, panel B shows the older-adult analysis retaining the Hutton-Arexvy estimate, and panel C shows the infant analysis, which was unchanged by the inclusion of Singer et al. CI, confidence interval; INB, incremental net benefit.*

*Figure S4. Supplementary subgroup analyses of incremental net benefit for RSV vaccination. Subgroup analyses stratified by target population and (A) region, (B) time horizon, (C) funding source, and (D) vaccine type. Panels A–C retained the Arexvy estimate from Hutton et al. In panel D, the Abrysvo and Arexvy estimates from Hutton et al. were included in their respective vaccine-type strata. CI, confidence interval; INB, incremental net benefit.*
