# Supplementary material for: Cost-effectiveness of respiratory syncytial virus vaccines in post-marketing settings: a systematic review and meta-analysis
Source: Infect Dis Poverty. 2026 Nov 14;15:117. doi: 10.1186/s40249-026-01520-y (PMC13770325; doi:10.1186/s40249-026-01520-y)
Supplement: Supplementary file 9 [file 40249_2026_1520_MOESM9_ESM.docx]

# Supplementary material 9. Meta-regression

Separate univariable random-effects meta-regression analyses were conducted for infants (*k* = 5) and older adults (*k* = 6) using the DerSimonian-Laird estimator. To avoid treating two correlated scenarios from the same study as independent observations, two older-adult scenario analyses were performed: Table S7 excluded Hutton-Arexvy and retained Hutton-Abrysvo, whereas Table S8 excluded Hutton-Abrysvo and retained Hutton-Arexvy. The moderators were vaccine price per dose, WTP, vaccine type, funding source, time horizon, and country/region. Among infants, only Asia versus Europe was statistically significant (β = -105.50, *P* < 0.001), while vaccine type was not estimable because all studies evaluated Abrysvo. In Table S7, Arexvy versus Abrysvo was statistically significant (β = 166.13, *P* = 0.045), whereas no moderator was statistically significant in Table S8. This difference indicates sensitivity to the selected Hutton scenario. Given the small number of studies, these study-level associations should be interpreted as exploratory.

**Table S6. Univariable meta-regression results for infants (*k* = 5)**

| **Variable/comparison** | **β ± SE** | ***P*** | **Residual *I*² (%)** |
| --- | --- | --- | --- |
| Vaccine price per dose (per unit increase) | 0.20 ± 0.55 | 0.721 | 92.04 |
| Willingness-to-pay (WTP) (per unit increase) | 0.0005 ± 0.0005 | 0.387 | 91.74 |
| Vaccine type (all studies: Abrysvo) | Not estimable | - | - |
| Funding source (manufacturer-funded vs non-manufacturer-funded) | 74.92 ± 49.50 | 0.130 | 90.36 |
| Time horizon (short-term vs long-term) | -5.50 ± 68.71 | 0.936 | 91.84 |
| Country/region (Asia vs Europe) | -105.50 ± 23.64 | < 0.001* | 43.37 |
| Country/region (United States vs Europe) | 51.56 ± 29.77 | 0.083 | 43.37 |

**Table S7. Older-adult meta-regression excluding Hutton-Arexvy (Hutton-Abrysvo retained; *k* = 6)**

| **Variable/comparison** | **β ± SE** | ***P*** | **Residual *I*² (%)** |
| --- | --- | --- | --- |
| Vaccine price per dose (per unit increase) | -1.37 ± 0.84 | 0.105 | 87.60 |
| Willingness-to-pay (WTP) (per unit increase) | -0.0007 ± 0.0008 | 0.378 | 89.12 |
| Vaccine type (Arexvy vs Abrysvo) | 166.13 ± 82.95 | 0.045* | 93.33 |
| Funding source (manufacturer-funded vs non-manufacturer-funded) | 158.47 ± 92.68 | 0.087 | 88.94 |
| Time horizon (short-term vs long-term) | -158.47 ± 92.68 | 0.087 | 88.94 |
| Country/region (United States vs Europe) | -32.61 ± 87.85 | 0.710 | 89.97 |

**Table S8. Older-adult meta-regression excluding Hutton-Abrysvo (Hutton-Arexvy retained; *k* = 6)**

| **Variable/comparison** | **β ± SE** | ***P*** | **Residual *I*² (%)** |
| --- | --- | --- | --- |
| Vaccine price per dose (per unit increase) | -1.52 ± 0.95 | 0.107 | 87.29 |
| Willingness-to-pay (WTP) (per unit increase) | -0.0007 ± 0.0008 | 0.361 | 89.14 |
| Vaccine type (Arexvy vs Abrysvo) | -3.17 ± 84.02 | 0.970 | 90.87 |
| Funding source (manufacturer-funded vs non-manufacturer-funded) | 162.22 ± 92.69 | 0.080 | 88.94 |
| Time horizon (short-term vs long-term) | -162.22 ± 92.69 | 0.080 | 88.94 |
| Country/region (United States vs Europe) | -35.12 ± 88.08 | 0.690 | 90.03 |

*Note: β is the univariable meta-regression coefficient and SE is its standard error. Residual I² quantifies heterogeneity remaining after inclusion of the moderator. Reference categories were Abrysvo, non-manufacturer funding, long-term time horizon, and Europe. Tables S7 and S8 differ only in which Hutton vaccine scenario was retained. *P < 0.05.*
